# Pancreatic adverse events of immune checkpoint inhibitors therapy for solid cancer patients: a systematic review and meta-analysis

**DOI:** 10.3389/fimmu.2023.1166299

**Published:** 2023-06-09

**Authors:** Zhe Zhao, Weike Zhang, Longbin Pang, Liangjie Zeng, Surui Liu, Jie Liu

**Affiliations:** ^1^ Department of Oncology, Jinan Central Hospital, Shandong University, Jinan, Shandong, China; ^2^ Pulmonary and Critical Care Medicine, Central Hospital Affiliated to Shandong First Medical University, Jinan, Shandong, China; ^3^ Department of Oncology, Central Hospital Affiliated to Shandong First Medical University, Jinan, Shandong, China

**Keywords:** pancreatic adverse events, drug-related adverse events, immune checkpoint inhibitors, immunotherapy, meta - analysis

## Abstract

**Objective:**

This review aims to determine the incidence and risk of pancreatic adverse events (AEs) associated with immune checkpoint inhibitors (ICIs) therapy for solid tumors.

**Methods:**

We conducted a comprehensive systematic literature search in PubMed, Embase, and Cochrane Library up to March 15, 2023, to identify all randomized controlled trials comparing ICIs with standard treatment in solid tumors. We included studies that reported immune-related pancreatitis or elevation of serum amylase or lipase levels. Following protocol registration in PROSPERO, we conducted a systematic review and meta-analysis.

**Results:**

59 unique randomized controlled trials with at least one ICI-containing arm (41 757 patients) were retrieved. The incidences for all-grade pancreatitis, amylase elevation and lipase elevation were 0.93% (95% CI 0.77-1.13), 2.57% (95% CI 1.83-3.60) and 2.78% (95% CI 1.83-4.19), respectively. The incidences for grade ≥3 pancreatitis, amylase elevation and lipase elevation were 0.68% (95% CI 0.54-0.85), 1.17% (95% CI 0.83-1.64) and 1.71% (95% CI 1.18-2.49), respectively. The use of ICIs was associated with an increased risk of all-grade pancreatic immune-related AEs (irAEs) including pancreatitis (OR=2.04, 95% CI 1.42-2.94, P =0.0001), amylase elevation (OR=1.91, 95% CI 1.47-2.49, P < 0.0001) and lipase elevation (OR=1.77, 95% CI 1.37-2.29, P < 0.0001). In addition to these, the *post-hoc* analysis found that PD-1 inhibitors had a significant higher risk of pancreatic AEs compared with PD-L1 inhibitors and the patients undergoing dual ICI therapy were at a significantly higher risk of pancreatic AEs than the patients receiving single ICI therapy.

**Conclusion:**

Our study provides an overview of the incidence and risk of ICI-associated pancreatitis and pancreatic enzyme elevations in the treatment of solid tumors. Our findings may help raise awareness among clinicians of the potential for ICI-associated pancreatic AEs in clinical practice.

**Systematic review registration:**

https://www.crd.york.ac.uk/PROSPERO, identifier 345350.

## Introduction

Immune checkpoint inhibitors (ICIs) including programmed cell death 1 (PD-1) inhibitors, programmed cell death 1 ligand 1(PD-L1)inhibitors and cytotoxic T- lymphocyte-associated antigen 4 (CTLA-4) inhibitors have revolutionized cancer therapy and become the standard treatment for a number of malignancies in the past few years (1, 2). While ICIs activate the immune system against tumor cells, they can also lead to adverse events due to the imbalance of immunologic homeostasis in normal tissues (3). IrAEs can range from mild self-limiting symptoms to severe life-threatening events that can affect nearly all organ systems. These adverse events include but are not limited to, colitis, hepatitis, dermatitis, pneumonia, endocrine disorders, nephritis, myocarditis, and neuropathy (4). As the use of immunotherapy in cancer patients continues to rise, uncommon irAEs present a significant clinical challenge (5). Pancreatic AEs are rare but often overlooked, requiring clinician attention due to their adverse impact on the quality of life of cancer patients.

Despite early clinical studies confirming the immune-related toxicity of ICIs in the pancreas (6), several questions remain unanswered. Firstly, how to effectively recognize pancreatic irAEs, as they may present as asymptomatic elevations in amylase and/or lipase levels, as per the guidelines of the National Comprehensive Cancer Network (NCCN) (7). Furthermore, it is unclear whether the incidence of pancreatic AEs increases with the widespread use of ICIs and whether different types of combination therapy affect the risk of incidence. Therefore, our study aims to address these knowledge gaps and provide insights into predicting and managing pancreatic irAEs through a systematic review and meta-analysis.

## Methods

### Search strategy

This systematic review and meta-analysis was conducted according to the Preferred Reporting Items for Systematic Reviews and Meta-Analysis (PRISMA) guidelines (8). The statement was registered at the International Prospective Register of Systematic Reviews (number 345350). We conducted a comprehensive systematic literature search in PubMed, Embase, and Cochrane Library up to March 15, 2023, for all randomized controlled trials(RCTs)that compared ICIs with standard treatment in solid tumors. Based on PICOS (participants, interventions, comparisons, outcomes, and study design) guidelines (9), the keywords and Medical Subject Headings (MeSH) terms were used as follows: “neoplasms”; “immune checkpoint inhibitor”, “PD-1 inhibitors”, “PD-L1 inhibitors”,” CTLA4 inhibitors” “pembrolizumab”, “nivolumab”, “tislelizumab”, “sintilimab”, “camrelizumab”, “toripalimab”, “atezolizumab”, “avelumab”, “durvalumab”, “cemiplimab”, “tremelimumab”, “Ipilimumab” “drug-related side effects and adverse reactions”, “adverse reactions”, and “randomized controlled trials”.

### Selection criteria

Studies eligible for inclusion met all the following criteria: (1) phase III RCTs including at least one ICI-containing arm (ICIs as monotherapy or in combination with another ICIs or standard treatment) in adult patients (age >_18 years) with solid cancer; (2) clinical trials reporting immune-related pancreatitis or elevation of serum amylase or lipase levels; and (3) studies published in English. The exclusion criteria were as follows: (1) studies published as abstracts, letters, or conference reports; (2) studies published repeatedly; (3)both treatment arms were immunotherapy.

### Data extraction

Two investigators (ZZ and WZ) independently evaluated the titles, abstracts, and full texts to select the potentially eligible publications. The following data were obtained from the included study: basic information (first author, publication year, trial name, and Clinical Trial number), participants(disease diagnosis, treatment arms, and the number of included patients), and the number of patients with pancreatitis, amylase elevation, and lipase elevation for all-grade (G1–5) and for grade 3 or higher (G3–5). The severity of the AE was graded on a scale from 0 to 5, with grade 0 being no toxicity and grade 5 being death according to the Common Terminology Criteria for Adverse Events(CTCAE) (10). Additional data included ICI regimen, control arm regimen, previous lines of chemotherapy, blindness, and median/mean follow-up (months). The primary outcome of our meta-analysis was the summary risk of pancreatic AEs associated with ICI exposure (ICIs as monotherapy or in combination with other ICIs or standard treatment) vs. controls in RCTs. If disagreement occurred, it was resolved by discussion with the corresponding author. All included studies represented unique trials.

### Statistical analysis

To conduct a meta-analysis of the incidence and profile of pancreatic AEs, a random effect model with logit transformation was applied. All models are fitted by restricted maximum likelihood estimation with a classic continuity correction of 0.5 for zero cells and the corresponding sample sizes. Multiple groups of a trial were combined separately. The outcome measure is the incidence with its 95% confidence interval (CI). Based on previous studies (11), we hypothesized that pancreatic AEs are not a frequent event (incidence < 10%), and we interpreted the odds ratio(OR) as a measure of risk (12, 13). Pooled ORs and 95% CIs were estimated with a random effects model using the Mantel–Haenszel method (14). If a study included more than one intervention arm, we separately compared each intervention arm with the control arm. In addition to that, we conducted subgroup analyses to examine studies by cancer type and combination type.

*Post-hoc* analyses were used to assess the pancreatic AEs differences between anti-PD-1 drugs and anti-PD-L1 drugs, as well as, between dual- and single -ICI therapies. We matched the included RCTs with their tumor type and intervention type, or tumor type and design of control groups to form several mirror groups for the adjusted indirect comparison (15). An OR (95% CI) was derived from each mirror group and then pooled across all ICI groups using a random-effects model.

We used the inconsistency index *ι^2^* statistic and χ2 test with its P-value to evaluate the heterogeneity between studies. According to the Cochrane Handbook for Systematic Reviews of Interventions, substantial heterogeneity between studies was defined by *ι^2^* value > 50%, and significant heterogeneity was defined by χ2 test P-value <0.10 (16). Publication bias was assessed using Peter tests with funnel plots, which is a recommended method for dichotomous data with low heterogeneity (17, 18). The risk of bias of included studies were evaluated with the Cochrane risk of bias tool (19). All analyses were done using Review Manager 5.3 software (Cochrane Collaboration 2014, Nordic Cochrane Center, Copenhagen, Denmark) and R statistical software (version 4.1.3; with the metafor_v3.0–2 packages) (20). A two-sided P-value of <0.05 in Z-tests (for overall effect) or χ2 test (for overall subgroup comparison) in all analyses was considered statistically significant.

## Results

### Eligible studies and characteristics

**Figure 1:**
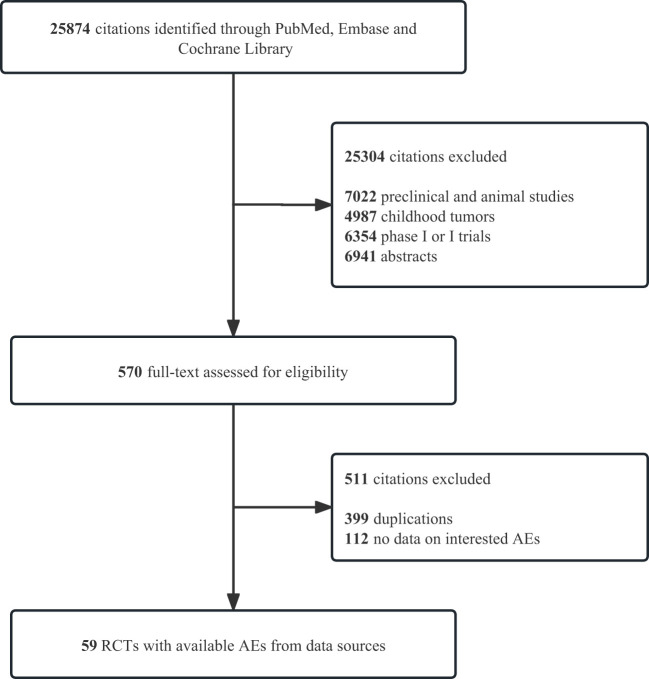
Study flow diagrams. PRISMA flow diagram of systematic review and meta-analysis in PubMed, Emabse, and Cochrane Library up to March 15, 2023. AEs, adverse events; RCTs, randomized controlled trials; PRISMA, Preferred Reporting Items for Systematic Reviews and Meta-Analyses.

We identified 25 874 records from PubMed, Embase, and Cochrane Library. **Figure 1** and **Supplementary Table 1** illustrate the details of the study screening and selection procedures. Finally, 59 eligible studies involving 41 757 patients for quantitative analysis were included. Details of the study characteristics are presented in **Table 1**. Among these 59 RCTs, one was a four-arm study and 9 RCTs were three-arm. The mean follow-up time for the entire population ranged from 7.3 to 41.2 months. According to the type of combination therapy, there were 30 arms of ICI monotherapy 32 arms of ICI plus chemotherapy or targeted therapy, and 8 arms of dual-ICI therapy. In our study, we incorporated multiple tumor types including non-small cell lung cancer (NSCLC, n =19) (21–39), small cell lung cancer(SCLC, n = 3) (40–42), melanoma (n = 6) (43–48), gastroesophageal junction cancer (GEJC, n = 6) (49, 51, 52, 54, 80, 81), urothelial carcinoma (UC, n = 4) (55–58), renal cell carcinoma (RCC, n=4) (59–62), breast cancer(BC,n=1) (63), head and neck squamous cell carcinoma (HNSCC,n=3) (64–66), prostate cancer (PC,n=1) (67), hepatocellular carcinoma (HCC,n=3) (68–70), esophageal carcinoma (ESO,n=2) (71, 72), ovarian cancer (OC,n=3) (73–75), colorectal cancer (CRC,n=1) (76), glioblastoma (n=1) (77) and mesothelioma (n=2) (78, 79). Among the 41 757 patients in the 59 trials that reported information on treatment-related deaths, no pancreatic-related deaths occurred. All included RCTs had a low risk of bias. A detailed evaluation of the risk of bias for each randomized controlled trial is presented in **Supplementary Table 2**.

**Table 1:** Characteristics of the randomized clinical trials included in the meta-analysis.

Study (Year)	Trial name (Clinical Trials.gov Identifier)	Type of cancer	Treatment arm	Patient (no.)	Pancreatitis (G1-5) (G3-5)		AMY (G1-5) (G3-5)		Lipase (G1-5) (G3-5)	
**D. Planchard** (2020) (21)	ARCTIC (NCT02352948)	NSCLC	Durvalumab	117	0	0	2	1	0	0
			Durvalumab+ Tremelimumab	173	2	2	4	0	0	0
			Tremelimumab	60	0	0	0	0	1	1
			Chemotherapy	110	0	0	0	0	0	0
**Martin Reck** (2019) (22)	KEYNOTE-024 (NCT02142738)	NSCLC	Pembrolizumab	154	1	1				
			Chemotherapy	150	0	0				
**Martin Reck** (2020) (23)	IMpower150 (NCT02366143)	NSCLC	Atezolizumab+ Bevacizumab + Chemotherapy	393	5					
			Atezolizumab+ Chemotherapy	400	2					
			Chemotherapy	394	0					
**Yi-Long Wu** (2019) (24)	CheckMate 078 (NCT02613507)	NSCLC	Nivolumab	337			1	1	1	1
			Chemotherapy	156			0	0	0	0
**Naiyer A. Rizvi** (2020) (25)	MYSTIC (NCT02453282)	NSCLC	Durvalumab	369			2	1	0	0
			Durvalumab+ Tremelimumab	371			3	3	3	3
			Chemotherapy	352			0	0	0	0
**Robert Jotte** (2020) (26)	IMpower131 (NCT02367794)	NSCLC	Atezolizumab+ Chemotherapy	666	3	2				
			Chemotherapy	334	0	0				
**Makoto** **Nishio**(2021) (27)	IMpower132 (NCT02657434)	NSCLC	Atezolizumab+ Chemotherapy	291	4	1				
			Chemotherapy	274	2	2				
**Yunpeng Yang**(2020) (28)	InnovENT (NCT03607539)	NSCLC	Sintilimab+ Chemotherapy	266			8	3		
			Chemotherapy	131			10	0		
**Enriqueta Felip**(2021) (29)	IMpower010 (NCT02486718)	NSCLC	Atezolizumab+ Chemotherapy	495	2	1				
			Chemotherapy	495	1	1				
**L. Gandhi** (2018) (30)	KEYNOTE-189 (NCT02578680)	NSCLC	Pembrolizumab +Chemotherapy	405	3	2				
			Chemotherapy	202	0	0				
**Howard West** (2019) (31)	IMpower130 (NCT02367781)	NSCLC	Atezolizumab+ Chemotherapy	473	2	0	0	0	1	1
			Chemotherapy	232	1	0	1	1	0	0
**Luis Paz-Ares** (2021) (32)	CheckMate 9LA (NCT03215706)	NSCLC	Nivolumab+ Ipilimumab + Chemotherapy	358	5	4	22	11	26	22
			Chemotherapy	349	0	0	6	0	4	3
**Ahmet Sezer** (2021) (33)	EMPOWER-Lung 1 (NCT03088540)	NSCLC	Cemiplimab	355			11	1	4	1
			Chemotherapy	342			2	1	0	0
**Tony S K Mok** (2019) (34)	KEYNOTE-042 (NCT02220894)	NSCLC	Pembrolizumab	636	1	0				
			Chemotherapy	615	0	0				
**Z. Wang** (2023) (35)	CHOICE (NCT03856422)	NSCLC	Toripalimab+ Chemotherapy	308	3	1	11	0		
			Chemotherapy	156	0	0	1	0		
**M. O'Brien** (2022) (36)	KEYNOTE-091 (NCT02504372)	NSCLC	Pembrolizumab	580	2	0	2	0	3	0
			Placebo	581	2	1	4	1	2	2
**M. Gogishvili** (2022) (37)	EMPOWER-Lung 3 (NCT034096614)	NSCLC	Cemiplimab+ Chemotherapy	312	1	0	22	3	15	1
			Chemotherapy	153	0	0	5	0	2	0
**G. de Castro** (2023) (38)	NEPTUNE (NCT02542293)	NSCLC	Durvalumab+ Tremelimumab	410	2	1				
			Chemotherapy	399	0	0				
**S. Peters** (2022) (39)	BFAST (NCT03178552)	NSCLC	Atezolizumab	234	2	1				
			Chemotherapy	221	1	0				
**Martin Reck** (2016) (40)	CA184-156 (NCT01450761)	SCLC	Ipilimumab+Chemotherapy	478			1	0	1	1
			Chemotherapy	476			0	0	0	0
**Charles M. Rudin** (2020) (41)	KEYNOTE-604 (NCT03066778)	SCLC	Pembrolizumab+ Chemotherapy	223	1	1				
			Chemotherapy	223	0	0				
**Jonathan** **W Goldman** (2021) (42)	CASPIAN (NCT03043872)	SCLC	Durvalumab+ Tremelimumab	266	2	1	6	1	10	6
			Durvalumab+ Chemotherapy	265	1	1	11	6	12	9
			Chemotherapy	266	0	0	2	1	7	4
**James Larkin** (2018) (43)	CheckMate 037 (NCT01721746)	Melanoma	Nivolumab	268	2					
			Chemotherapy	102	0					
**Antoni Ribas** (2013) (44)	(NCT00257205)	Melanoma	Tremelimumab	328	3	3				
			Chemotherapy	327	0	0				
**Ralf Gutzmer** (2020) (45)	IMspire150 (NCT02908672)	Melanoma	Atezolizumab+ Vemurafenib + Cobimetinib	230	5	0	46	23	74	47
			Vemurafenib+ Cobimetinib	281	1	0	45	19	77	58
**Jeffff rey S Webe** (2015) (46)	CheckMate 037 (NCT01721746)	Melanoma	Nivolumab	268						3
			Chemotherapy	102						1
**M. B. Atkins** (2023) (47)	EA6134 (NCT02224781)	Melanoma	Nivolumab+ Ipilimumab	126	2	1	13	7	18	13
			Dabrafenib+Trametinib	130	0	0	12	1	22	7
**G. V. Long** (2022) (48)	KEYNOTE-716 (NCT03553836)	Melanoma	Pembrolizumab	487	2	2	3	1	6	4
			Placebo	489	0	0	1	1	8	2
**Y.-J. Bang** (2018) (49)	JAVELIN Gastric 300 (NCT02625623)	GEJC	Avelumab	184					1	1
			Chemotherapy	177					2	2
**Markus Moehler** (2020) (50)	JAVELIN Gastric 100 (NCT02625610)	GEJC	Avelumab	243			11	2	9	2
			Chemotherapy	238			9	4	14	7
**Kohei Shitara** (2020) (51)	KEYNOTE-062 (NCT02494583)	GEJC	Pembrolizumab	254	2					
			Pembrolizumab +Chemotherapy	250	0					
			Chemotherapy	244	1					
**Yelena Y Janjigian** (2021) (52)	CheckMate 649 (NCT02872116)	GEJC	Nivolumab+ Chemotherapy	782					89	45
			Chemotherapy	767					34	16
**Yoon-Koo Kang** (2021) (53)	ATTRACTION-4 (NCT02746796)	GEJC	Nivolumab+ Chemotherapy	359			1	0		
			Chemotherapy	358			4	1		
**Kohei Shitara** (2018) (54)	KEYNOTE-061 (NCT02370498)	GEJC	Pembrolizumab	294	0	0				
			Chemotherapy	276	1	1				
**D.F. Bajorin** (2021) (55)	CheckMate 274 (NCT02632409)	UC	Nivolumab	351			33	13	34	18
			Placebo	348			20	5	20	9
**Joaquim** **Bellmunt** (2021) (56)	IMvigor010 (NCT02450331)	UC	Atezolizumab	390	2	1	5	2	5	3
			Placebo	397	2	2	0	0	0	0
**Thomas Powles** (2020) (57)	DANUBE (NCT02516241)	UC	Durvalumab	345	1	0	9	3	11	7
			Durvalumab+ Chemotherapy	340	5	3	12	8	20	16
			Chemotherapy	313	2	1	1	0	2	1
**Thomas Powles** (2021) (58)	KEYNOTE-361 (NCT02853305)	UC	Pembrolizumab+ Chemotherapy	349	2	2		12		2
			Pembrolizumab	302	2	2		0		0
			Chemotherapy	342	0	0		0		0
**R.J. Motzer** (2018) (59)	CheckMate 214 (NCT02231749)	RCC	Nivolumab+ Ipilimumab	547					90	56
			Sunitinib	535					58	35
**T.K. Choueiri** (2021) (60)	CheckMate 9ER (NCT03141177)	RCC	Nivolumab + Cabozantinib	320			47	10	53	20
			Sunitinib	320			29	8	38	15
**Thomas Powles**(2020) (61)	KEYNOTE-426 (NCT02853331)	RCC	Pembrolizumab+ Axitinib	429	5	4				
			Sunitinib	425	3	3				
**S. K. Pal** (2022) (62)	IMMOTION-010 (NCT03024996)	RCC	Atezolizumab	390	1	0	4	1	1	1
			Placebo	383	1	1	2	0	3	2
**Elizabeth A Mittendorf** (2020) (63)	IMpassion031 (NCT03197935)	BC	Atezolizumab+ Chemotherapy	164	0	0				
			Chemotherapy	167	0	0				
**Barbara Burtness** (2019) (64)	KEYNOTE-048 (NCT02358031)	HNSCC	Pembrolizumab	300	2	0				
			Pembrolizumab+ Chemotherapy	276	1	1				
			Cetuximab + Chemotherapy	287	0	0				
**Ezra E W Cohen** (2019) (65)	KEYNOTE-040 (NCT02252042)	HNSCC	Pembrolizumab	246			1	1		
			Chemotherapy	234			0	0		
**Nancy Y Lee** (2021) (66)	JAVELIN Head and Neck 100 (NCT02952586)	HNSCC	Avelumab+ Chemotherapy	348			20	6	10	5
			Chemotherapy	344			5	2	5	1
**Eugene D Kwon** (2014) (67)	CA184-043 (NCT00861614)	PC	Ipilimumab	393			2	2	2	1
			Placebo	396			1	0	2	2
**Zhenggang Ren** (2021) (68)	ORIENT-32 (NCT03794440)	HCC	Sintilimab + Bevacizumab	380			2			
			Sorafenib	185			0			
**A. L. Cheng** (2022) (69)	IMbrave-150 (NCT03434379)	HCC	Atezolizumab+ Bevacizumab	329	10	4				
			Sorafenib	156	6	5				
**R. K. Kelley** (2022) (70)	COSMIC-312 (NCT03755791)	HCC	Atezolizumab+ Cabozantinib	429	4	3	24	3	28	7
			Sorafenib	395	2	0	14	1	14	5
**Jing Huang** (2020) (71)	ESCORT (NCT03099382)	ESO	Camrelizumab	228	1	1				
			Chemotherapy	220	0	0				
**Jong-Mu Sun** (2021) (72)	KEYNOTE-590 (NCT03189719)	ESO	Pembrolizumab+ Chemotherapy	370	2	0				
			Chemotherapy	370	1	1				
**Kathlen N. Moore** (2021) (73)	IMagyn050 (NCT03038100)	OC	Atezolizumab+ Bevacizumab+ Chemotherapy	642	5	4				
			Bevacizumab+ Chemotherapy	644	0	0				
**Eric Pujade-Lauraine** (2021) (74)	JAVELIN Ovarian 200 (NCT02580058)	OC	Avelumab+ Chemotherapy	182			5	1	3	2
			Avelumab	187			3	0	1	1
			Chemotherapy	177			1	1	0	0
**Bradley J Monk** (2021) (75)	JAVELIN Ovarian 100 (NCT02718417)	OC	Avelumab+ Chemotherapy	657	1	1	13	4	18	13
			Chemotherapy	334	0	0	4	1	3	2
**Cathy Eng** (2019) (76)	IMblaze 370 (NCT02788279)	CRC	Atezolizumab+ Cobimetinib	179	2	2	6	3	9	4
			Atezolizumab	90	1	1	2	0	1	1
			Regorafenib	80	0	0	3	0	6	1
**D.Reardon** (2020) (77)	CheckMate 143 (NCT02017717)	Glioblastoma	Nivolumab	182			3	2	7	4
			Bevacizumab	165			1	0	1	0
**Paul Baas** (2021) (78)	CheckMate 743 (NCT02899299)	Mesothelioma	Nivolumab+ Ipilimumab	300	2	0	17	7	20	13
			Chemotherapy	284	0	0	1	0	1	1
**Dean AFennel** (2021) (79)	CONFIRM (NCT03063450)	Mesothelioma	Nivolumab	221	1				1	1
			Placebo	111	0				0	0

AMY, amylase elevation; Lipase, lipase elevation; G1–5, grade1–5; G3–5, grade3–5.

### Incidence of pancreatic AEs

A total of 41 757 patients were enrolled in the 59 included RCTs (70 ICI-containing arms), including 23 334 (55.9%) patients in the ICI-containing arms and 18 423 patients in the control arms (44.1%). ICI-containing arms included ICI monotherapy in 30/70 arms, ICI plus chemotherapy or targeted therapy in 32/70 arms, and ICI dual therapy in 8/70 arms. In the included 70 arms, NSCLC was the most common tumor type, accounting for 32.9% (23/70), and GEJC accounted for 10.0% (7/70) as the second most common type.

**Figure 2:**
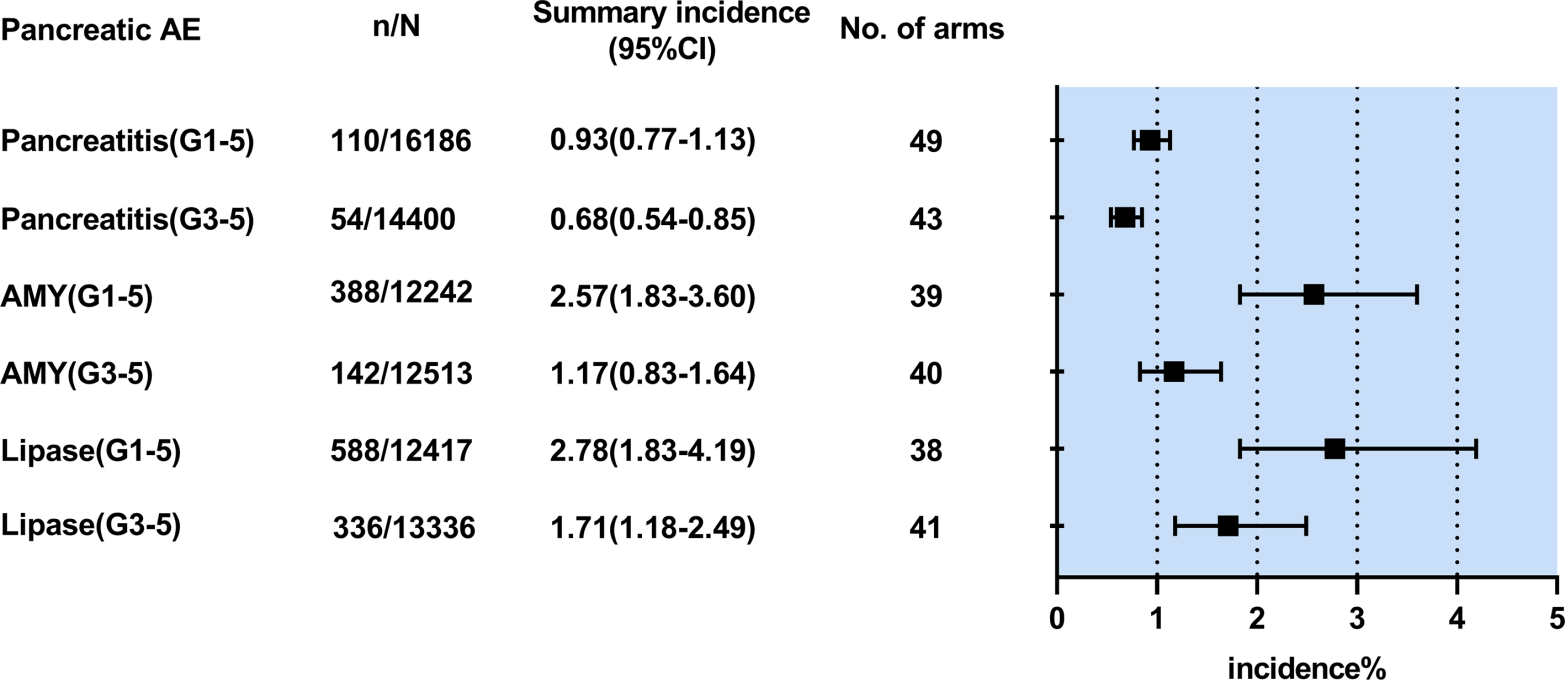
Summary pooled incidence analysis of pancreatic adverse events associated with immune checkpoint inhibitor therapy. n/N refers to the number of events (n) observed for the outcome regarding the overall number of patients (N) in patients treated with immune checkpoint inhibitor therapy. AE,adverse event; CI, confidence interval; AMY,amylase elevation;Lipase, lipase elevation;G1-5,grade1-5;G3-5.grade3-5.

The incidence was 0.93% (95% CI 0.77-1.13, *I²*=3.4%) for all-grade pancreatitis and 0.68% (95% CI 0.54-0.85; *I²*=0) for grade ≥3 pancreatitis. (**Figure 2**) Compared with ICI monotherapy, dual-ICI therapy had significantly higher incidences of all-grade pancreatitis (1.10% vs 0.70%) and grade ≥3 pancreatitis (0.94% vs 0.58%) (P< 0.05). (**Supplementary Table 3**) However, it was not observed in the patients undergoing ICI plus chemotherapy or targeted therapy. An overview of the pancreatitis incidence in different tumor types was shown in **Supplementary Table 3**. Pancreatitis has a roughly similar incidence in different tumor types (G1-5: 0.30-1.79%, G3-5: 0.17-1.12%).

The incidence was 2.57% (1.83-3.60; *I²*=89.2%) for all-grade amylase elevation and 1.17% (0.83-1.64; *I^2 =^* 76%) for grade ≥3 amylase elevation. (**Figure 2**) Compared with ICI monotherapy, dual-ICI therapy had significantly higher incidences of all-grade amylase elevation (3.01% vs 1.66%) and grade ≥3 amylase elevation (1.79% vs 0.78%) (P< 0.05). (**Supplementary Table 4**)Similar results were found in the patients treated with ICI plus chemotherapy or targeted therapy (G1-5: 3.78% vs 1.66%, G3-5: 1.57% vs 0.78%, P< 0.05). An overview of the amylase elevation incidence in different treatment regimens and tumor types is shown in **Supplementary Table 4**. The results showed an increased incidence of all-grade and grade ≥3 amylase elevation in patients with melanoma (5.62%,2.75% respectively) and Mesothelioma (5.67%, 2.33% respectively).

The incidence was 2.78% (1.83-4.19, *I^2 =^* 93%) for all-grade lipase elevation and 1.71% (1.18-2.49, *I^2 =^* 89%) for grade ≥3 lipase elevation. (**Figure 2**) Compared with ICI monotherapy, dual-ICI therapy had significantly higher incidences of all-grade lipase elevation (4.08% vs 1.45%) and grade ≥3 lipase elevation (3.28% vs 1.01%) (P< 0.05). (**Supplementary Table 5**)We found similar outcomes in the patients receiving ICI plus chemotherapy or targeted therapy compared with ICI monotherapy (G1-5: 5.34% vs 1.45%, G3-5: 2.23% vs 1.01%, P< 0.05). An overview of the amylase elevation incidences in different treatment regimens and tumor types is shown in **Supplementary Table 5**. The patients with melanoma (9.28%,6.14%) are most likely to develop all-grade and grade ≥3 lipase elevation.

### Risk of pancreatitis associated with ICI exposure

Pancreatitis as a treatment-related adverse effect was reported in 40 studies (49 ICI-containing arms) and graded using CTCAE. A total of 28 097 patients were evaluated with 16 186 in the ICI-containing arms and 11 911 in the control arms. As shown in **Table 2**, ICIs significantly increased the risk of all-grade pancreatitis (OR=2.04, 95% CI 1.42-2.94, P = 0.0001; *I²*=0) and grade ≥3 pancreatitis (OR=1.90, 95% CI 1.15-3.13, P=0.01; *I²*=0). Subgroup analysis suggested that dual-ICI therapy was associated with a higher incidence risk of all-grade pancreatitis (OR=3.47, 95%CI 1.22-9.91, P=0.02). (**Supplementary Table 6**) A similar statistically significant difference was found in grade ≥3 pancreatitis(OR=3.56, 95%CI 1.09-11.56, P=0.04). Tumor type-stratified analyses showed an increased risk of all-grade pancreatitis(OR=2.55, 95%CI 1.32-4.92, P=0.005) in patients with NSCLC.

**Table 2:** Summary pooled analysis on the risk of ICI therapy-associated pancreatic adverse events vs. controls in randomized controlled trials.

Variables	Pancreatic AEs							
	Grade 1-5				Grade 3-5			
	OR	95%CI	P	I²	OR	95%CI	P	I²
**Pancreatitis**	2.04	1.42-2.94	P=0.0001	0	1.90	1.15-3.13	P=0.01	0
**Amylase Elevation**	1.91	1.47-2.49	P<0.0001	29%	2.04	1.46-2.85	P=0.0001	0
**Lipase Elevation**	1.77	1.37-2.29	P<0.0001	45%	1.89	1.45-2.45	P<0.0001	18%

ICI, immune checkpoint inhibitor; AEs, adverse events; CI, confidence interval; OR odds ratio.

### Risk of amylase elevation associated with ICI exposure

Amylase elevation as a treatment-related adverse effect was reported in 33 studies (41 ICI-containing arms) and graded using CTCAE. A total of 22 390 patients were evaluated with 12 893 in the ICI-containing arms and 9 497 in the control arms. As shown in **Table 2**, ICIs significantly increased the risk of all-grade amylase elevation (OR=1.91, 95% CI 1.47-2.49, P < 0.0001; *I²*= 29%) and grade ≥3 amylase elevation (OR=2.04, 95% CI 1.46-2.85, P=0.0001; *I²* = 0). Subgroup analysis suggested that all three therapies that include ICI could significantly increase the incidence risk of all-grade amylase elevation (OR=1.86, 95% CI 1.28-2.69, P=0.001; OR=1.60, 95% CI 1.09-2.35, P=0.02 and OR=3.79, 95% CI 1.68-8.57, P=0.001, respectively). (**Supplementary Table 7**) Tumor type-stratified analyses showed an increased risk of all-grade amylase elevation in patients with SCLC (OR=4.10,95% CI 1.44-11.63, P=0.008), UC (OR=4.64,95% CI 1.30-16.49, P=0.02), RCC (OR=1.71,95% CI 1.06-2.74, P=0.03), HNSCC (OR=4.00,95% CI 1.55-10.33, P=0.004) and mesothelioma (OR=17.00,95% CI 2.25-128.60, P=0.006).

### Risk of lipase elevation associated with ICI exposure

Lipase elevation as a treatment-related adverse effect was reported in 32 studies (40 ICI-containing arms) and graded using CTCAE. A total of 23 461 patients were evaluated with 13 336 in the ICI-containing arms and 10 125 in control arms. As shown in **Table 2**, ICIs significantly increased the risk of all-grade lipase elevation (OR=1.77, 95% CI 1.37-2.29, P < 0.0001; *I²*= 45%) and grade ≥3 lipase elevation (OR=1.89, 95% CI 1.45-2.45, P< 0.0001; *I²* = 18%). Subgroup analysis suggested that both ICI plus chemotherapy or targeted therapy and dual-ICI therapy could significantly increase the incidence risk of all-grade lipase elevation (OR=1.72, 95% CI 1.34-2.20, P<0.0001, and OR=2.92, 95% CI 1.37-6.20, P=0.005 respectively). (**Supplementary Table 8**) As for grade ≥3 lipase elevation, the trends are similar to those of the all-grade lipase elevation groups. At the same time, we observed a significant increase in the risk of all-grade lipase elevation in the patient with NSCLC (OR=4.23,95% CI 2.14-8.34, P<0.0001), UC (OR=4.20,95% CI 1.46-12.09, P=0.008), RCC (OR=1.53,95% CI 1.16-2.01, P=0.003), and OC (OR=3.42,95% CI 1.17-9.97, P=0.02).

### *Post-hoc* analyses

In this study, we conducted *post-hoc* analyses of PD-1/PD-L1 inhibitors related to pancreatic AEs. As shown in **Table 3**, the patients with UC undergoing PD-1 inhibitors were at a significantly higher risk of all-grade amylase elevation (OR=5.24,95% CI 2.59-10.57, P<0.0001), all-grade lipase elevation (OR=4.90,95% CI 1.97-12.18, P=0.0006) and grade ≥3 lipase elevation (OR=3.88,95% CI 1.50-10.04, P=0.005), than the patients with UC receiving PD-L1 inhibitors. We conducted *post-hoc* analyses of dual ICI therapy/single ICI therapy-related pancreatic AEs. As shown in **Table 4**, the patients with NSCLC undergoing dual ICI therapy were at a significantly higher risk of all-grade pancreatitis (OR=4.72,95% CI 1.11-20.17, P=0.04), grade ≥3 pancreatitis (OR= 14.98,95% CI 1.82-123.34, P= 0.01), grade ≥3 amylase elevation (OR=5.95,95% CI 1.30-27.24, P=0.02) and all-grade lipase elevation (OR=4.99,95% CI 1.99-12.55, P=0.0006), than the patients with NSCLC receiving single ICI therapy.

**Table 3:** Odds ratios comparing pancreatic irAEs in patients who received anti-PD-1- vs anti-PD-L1-based therapies.

Cancer	Pancreatitis				Amylase Elevation				Lipase Elevation			
	Grade 1-5		Grade3-5		Grade 1-5		Grade3-5		Grade 1-5		Grade3-5	
	OR (95%CI)	P	OR (95%CI)	P	OR (95%CI)	P	OR (95%CI)	P	OR (95%CI)	P	OP (95%CI)	P
**NSCLC**	1.25 (0.61-2.55)	0.55	2.15 (0.71-6.52)	0.18	3.05 (0.20-45.65)	0.42	1.57 (0.19-12.78)	0.68	3.47 (0.39-31.17)	0.27	1.81 (0.19-17.47)	0.61
**SCLC**	1.14 (0.07-18.29)	0.09	1.14 (0.07-18.29)	0.93	–	–	–	–	–	–	–	–
**UC**	0.84 (0.27-2.56)	0.76	1.45 (0.39-5.32)	0.58	5.24 (2.59-10.57)	<0.0001	1.74 (0.34-8.83)	0.50	4.90 (1.97-12.18)	0.0006	3.88 (1.50-10.04)	0.005

irAEs, immune-related adverse events; OR, odds ratio; CI, confidence interval; NSCLC, non-small cell lung cancer; SCLC, small cell lung cancer; UC, urothelial carcinoma; Total, pan-cancer.

**Table 4:** Odds ratios comparing pancreatic irAEs in patients who received dual ICI therapy - vs single ICI therapy -based therapies.

Cancer	Pancreatitis				Amylase Elevation				Lipase Elevation			
	Grade 1-5		Grade3-5		Grade 1-5		Grade3-5		Grade 1-5		Grade3-5	
	OR (95%CI)	P	OR (95%CI)	P	OR (95%CI)	P	OR (95%CI)	P	OR (95%CI)	P	OP (95%CI)	P
**NSCLC**	4.72 (1.11-20.17)	0.04	14.98 (1.82-123.34)	0.01	2.98 (0.97-9.16)	0.06	5.95 (1.30-27.24)	0.02	4.99 (1.99-12.55)	0.0006	4.91 (0.69-35.02)	0.11

irAEs, immune-related adverse events; OR, odds ratio; CI, confidence interval; NSCLC, non-small cell lung cancer.

### Quality of included studies

Given the significant heterogeneity in the meta-analysis of all the included studies, we performed subgroup analyses to better understand the heterogeneity. (**Supplementary Table 9**) Some study heterogeneity was suggested by the assessment of all-grade amylase elevation (*I²* = 36%), which appeared to be concentrated in the studies of NSCLC (*I²* = 59%), GJEC (*I²* = 42%) and UC (*I²* = 54%). A similar situation could also be observed with the group of all-grade lipase elevation (*I²*=46%) and grade 3 or higher lipase elevation (*I²*=26%).

No obvious asymmetry was seen in classic funnel plots, indicating that no evidence of significant publication bias existed. Beyond this, the above view was confirmed by Peter’s test. (**Supplementary Table 10**).

## Discussion

In our meta-analysis, we investigated the incidence and risk of pancreatic irAEs associated with ICIs, including pancreatitis, amylase elevation, and lipase elevation. Our findings demonstrated that the incidence of all-grade and grade≥3 pancreatitis with ICIs were 0.93% and 0.68%, respectively. These rates were consistent with previous studies reporting rates of pancreatitis (CTLA-4: 0.9–3%, PD-1: 0.5–1.6%, CTLA4 + PD-1: 1.2–2.1%) (11). Our results also showed that patients treated with dual ICIs therapy had a higher incidence of pancreatitis compared to those treated with monotherapy, and the combination of ICI monotherapy with chemotherapy, targeted therapy, or immunotherapy increased the incidence of pancreatic enzyme elevation. Moreover, our study revealed that melanoma patients had the highest incidence of amylase elevation (G1-5: 5.62%, G3-5: 2.75%) and all-grade and grade 3 or higher lipase elevation (G1-5: 9.28%, G3-5: 6.14%) after receiving immunotherapy.

Our study findings revealed a significant increase in the incidence of pancreatitis, regardless of all grades or grades 3-5, in the ICI group compared to standard chemotherapy or targeted therapy. Further, our subgroup analysis identified a tumor-specific preference for pancreatitis, which was more likely to occur in HCC. Our data suggested that ICI monotherapy did not increase the risk of immune-related pancreatitis, whereas ICI combination therapy did. This may be attributed to the potential of chemotherapeutic agents and targeted drugs to exacerbate pancreatic damage from ICIs. Notably, our data indicated a higher likelihood of pancreatitis in the ICI dual therapy group (G1-5: OR=3.47, 95% CI 1.22-9.91, P=0.02; G3-5: OR=3.56, 95% CI 1.09-11.65, P=0.04). Therefore, additional multi-center RCTs are warranted to confirm its statistical significance. Our results align with previous studies (11, 82, 83).

According to many experts, pancreatitis is more likely to occur in the early stages with low grades, but can be controlled with aggressive intravenous fluid replacement (84, 85). Routine monitoring of amylase and lipase is not recommended for asymptomatic patients unless pancreatitis is clinically suspected (85). However, one study suggests that the use of ICI may increase the risk of developing grade 3 or higher pancreatitis, with clinical symptoms including loss of appetite, vomiting, and abdominal pain (86). Additionally, a case report described a 65-year-old man with stage IV melanoma who developed grade 3 pancreatitis while receiving ipilimumab and pembrolizumab (87). Despite the resolution of clinical signs and symptoms, the patient was diagnosed with pancreatic insufficiency. Interestingly, it seemed that diabetes was also associated with pancreatitis. One study showed that both immune-related pancreatitis and immune-related diabetes occurred earlier than monotherapy when two ICIs were combined, and immune-related diabetes had a later onset than immune-related pancreatitis (88), suggesting that the onset of diabetes might also be a complication of immune-related pancreatitis (89). In order to improve the quality of life and to avoid the long-term sequelae of pancreatitis in patients who have used ICI, vigilant monitoring should be warranted (90).

So far, the exact mechanism of immune-related pancreatitis remains under investigation, and the potential mechanisms may include the increased activity of T cells against antigens present on tumors and normal tissues,the increase in the concentration of pre-existing autoimmune antibodies and the increased levels of inflammatory cytokines (91). Immunohistochemical staining demonstrated a large infiltration of CD3+ T lymphocytes in the non-tumor regions of the pancreas from patients with immune-related pancreatitis (92, 93), which suggested that the potential asscociation of immune-related pancreatitis with autoimmune pancreatitis (AIP) (94). The clinical presentation of AIP differred from that of acute pancreatitis in that abdominal pain and nausea was milder, and positive imaging might be delayed (95).

It is worth noting that despite their widespread use, steroids were not found to be effective in treating immune-related pancreatitis in terms of preventing short- or long-term adverse outcomes, or improving overall survival (84). In fact, exposure to a baseline dose of prednisone equivalent to at least 10 mg/d was found to reduce the efficacy benefit of ICI and significantly shorten progression-free survival (PFS) and overall survival (OS) in NSCLC patients (96). Patients with immune-related pancreatitis were reported to be at risk of relapse upon the resumption of ICI therapy (97). Nonetheless, in general, immunotherapy may be resumed when toxicity returns to grade 1 or lower (85). Our study found that amylase and lipase elevations were more frequent in the ICI group, suggesting a potential immune-related mechanism. Subgroup analyses revealed a significantly higher incidence of all-grade amylase and lipase elevations in melanoma patients. The tumor-specific preference for immune-related elevation of pancreatic enzymes and pancreatitis was similar, with both showing a predilection for NSCLC and UC, as demonstrated by grouping methods based on tumor type or ICI regimen. However, non-specific elevations of pancreatic enzymes due to factors such as alcohol consumption, bowel obstruction, or kidney failure may also occur, leading to a potential overestimation of the incidence of immune-related elevations (98, 99). Nevertheless, unlike pancreatitis, our study provided compelling evidence of a plausible causal association between ICI therapy and elevations of amylase and lipase. We hypothesized that ICI therapy may result in weak pancreatic injury, such as enzyme elevations, rather than robust injury like immune-related pancreatitis. Nonetheless, the decision to continue ICI therapy in patients with grade 3 or higher amylase or lipase elevations without clinical or imaging evidence of pancreatitis after immunotherapy requires further investigation.

It is assumed that the elevation of pancreatic enzymes is associated with pancreatitis and could implicate its development. Research has shown that elevated amylase levels increase the risk of pancreatitis (100). Additionally, 39% of patients with grade 3 or higher lipase elevations had significant clinical symptoms of pancreatitis (84), which was consistent with a retrospective study of 21 cases of immune-related lipase elevations (101). Patients with clinically symptomatic immune-related pancreatitis had higher mean peak serum lipase levels than those without clinical symptoms, but this was not the case in patients with other causes of acute pancreatitis (100). These studies demonstrated that elevated pancreatic enzyme values do not determine the severity of pancreatitis but indicate an increased risk. However, another study found that the true incidence of pancreatitis in patients with immune-related lipase elevations was only 14%, suggesting that in patients with elevated immune-related lipase without clinical symptoms, pancreatic X-ray abnormalities, and diabetes mellitus by fasting blood glucose, the lipase increase may be regarded as a non-clinically significant event (101). Further clinical trials are needed to confirm these findings.

In the *post-hoc* analysis, the findings indicated that PD-1 inhibitors had a significantly higher risk of pancreatic AEs compared to PD-L1 inhibitors, consistent with other immune-related adverse events, such as pneumonitis (15). Furthermore, the study revealed a statistically significant increase in the incidence of pancreatic AEs with dual-ICI therapy relative to single-ICI therapy, possibly due to the similarity in toxicity profiles of CTLA-4 inhibitors and PD-1 inhibitors. In Phase II and III trials of patients with nonresectable melanoma who were randomized to combination versus monotherapy, grade 3 or 4 adverse events occurred in 55–59% of the patients receiving combination therapy, as compared with 16–21% with nivolumab alone and 27–28% with ipilimumab alone (102, 103). Therefore, it is important to be vigilant about the occurrence of irAEs when using dual-ICI therapy, including monitoring pancreatic enzymes.

The study had several limitations. Firstly, our meta-analysis was based on phase III RCTs with strict inclusion criteria, which may limit the generalizability of the findings to real-world settings. Secondly, we may have missed some pancreatic AE cases, as we only analyzed cases recorded in the main text and appendix, which could result in reporting bias (104). Furthermore, some studies included in the analysis were open-label. Thirdly, individual patient data was not available, which prevented us from analyzing the relationship between pancreatic enzyme elevations and pancreatitis or linking immune-related pancreatitis with other irAEs. Lastly, although we acknowledged that drug dose might affect the incidence of irAEs, we were unable to conduct subgroup analyses due to the wide variation in drugs and doses across studies.

## Conclusion

Our study offers a comprehensive overview of the incidence and risk of ICI-associated pancreatitis and pancreatic enzyme elevations in various solid tumor types and treatment combinations. Moreover, the *post-hoc* analysis revealed that PD-1 inhibitors have a significantly higher risk of pancreatic AEs than PD-L1 inhibitors, and patients receiving dual ICI therapy have a significantly higher risk of pancreatic AEs than those receiving single ICI therapy. These findings should enhance clinicians’ awareness of ICI-associated pancreatic AEs in their clinical practice.

## Data availability statement

The original contributions presented in the study are included in the article/**Supplementary Material**. Further inquiries can be directed to the corresponding author.

## Author contributions

JL, ZZ, and LP designed the search strategy and confirmed the inclusion criteria. ZZ, WZ, LZ, and SL searched the database, selected the articles, and collected the data. ZZ, LP, WZ, LZ, and SL completed the quality assessment that JL checked. ZZ, LP, WZ, LZ, and SL finished data synthesis and statistics. ZZ write-original draft preparation. JL revised the manuscript carefully. All authors contributed to the article and approved the submitted version.

## References

[1] Ribas A Wolchok JD . Cancer immunotherapy using checkpoint blockade. Science (2018) 359(6382):1350–5. doi: 10.1126/science.aar4060 PMC7391259 29567705

[2] Tang J Shalabi A Hubbard-Lucey V . Comprehensive analysis of the clinical immuno-oncology landscape. Ann Oncol (2018) 29(1):84–91. doi: 10.1093/annonc/mdx755 29228097

[3] Tirumani SH Ramaiya NH Keraliya A Bailey ND Ott PA Hodi FS . Radiographic profiling of immune-related adverse events in advanced melanoma patients treated with ipilimumab. Cancer Immunol Res (2015) 3(10):1185–92. doi: 10.1158/2326-6066.CIR-15-0102 PMC4596761 26100356

[4] Darnell EP Mooradian MJ Baruch EN Yilmaz M Reynolds KL . Immune-related adverse events (irAEs): diagnosis, management, and clinical pearls. Curr Oncol Rep (2020) 22(4):39. doi: 10.1007/s11912-020-0897-9 32200442

[5] Postow MA Callahan MK Wolchok JD . Immune checkpoint blockade in cancer therapy. J Clin Oncol (2015) 33(17):1974–82. doi: 10.1200/JCO.2014.59.4358 PMC4980573 25605845

[6] Wang PF Chen Y Song SY Wang TJ Ji WJ Li SW . Immune-related adverse events associated with anti-PD-1/PD-L1 treatment for malignancies: a meta-analysis. Front Pharmacol (2017) 8:730. doi: 10.3389/fphar.2017.00730 29093678 PMC5651530

[7] Thompson JA . New NCCN guidelines: recognition and management of immunotherapy-related toxicity. J Natl Compr Canc Netw (2018) 16(5s):594–6. doi: 10.6004/jnccn.2018.0047 29784734

[8] Moher D Liberati A Tetzlaff J Altman DG . Preferred reporting items for systematic reviews and meta-analyses: the PRISMA statement. Ann Internal Med (2009) 151(4):264–9, w64. doi: 10.1371/journal.pmed.1000097 19622511

[9] Amir-Behghadami M Janati A . Population, intervention, comparison, outcomes and study (PICOS) design as a framework to formulate eligibility criteria in systematic reviews. Emerg Med J (2020) 37(6):387. doi: 10.1136/emermed-2020-209567 32253195

[10] Cancer therapy evaluation program (CTEP) . Available at: https://ctep.cancer.gov/.

[11] Su Q Zhang XC Zhang CG Hou YL Yao YX Cao BW . Risk of immune-related pancreatitis in patients with solid tumors treated with immune checkpoint inhibitors: systematic assessment with meta-analysis. J Immunol Res (2018) 2018:1027323. doi: 10.1155/2018/1027323 29971244 PMC6008648

[12] Sedgwick P . Relative risks versus odds ratios. BMJ (Online) (2014) 348(feb07 2):g1407. doi: 10.1136/bmj.g1407

[13] Ranganathan P Aggarwal R Pramesh CS . Common pitfalls in statistical analysis: odds versus risk. Perspect Clin Res (2015) 6(4):222–4. doi: 10.4103/2229-3485.167092 PMC4640017 26623395

[14] DerSimonian R Laird N . Meta-analysis in clinical trials revisited. Contemp Clin Trials (2015) 45(Pt A):139–45. doi: 10.1016/j.cct.2015.09.002 PMC4639420 26343745

[15] Duan J Cui L Zhao X Bai H Cai S Wang G . Use of immunotherapy with programmed cell death 1 vs programmed cell death ligand 1 inhibitors in patients with cancer: a systematic review and meta-analysis. JAMA Oncol (2020) 6(3):375–84. doi: 10.1001/jamaoncol.2019.5367 PMC6990765 31876895

[16] Higgins JPT Thomas J Chandler J Cumpston M Li T Page MJ . Cochrane handbook for systematic reviews of interventions version 6.3 (updated February 2022). Cochrane (2022). Available at: www.training.cochrane.org/handbook.

[17] Harbord RM Egger M Sterne JA . A modified test for small-study effects in meta-analyses of controlled trials with binary endpoints. Stat Med (2006) 25(20):3443–57. doi: 10.1002/sim.2380 16345038

[18] Sterne JA Sutton AJ Ioannidis JP Terrin N Jones DR Lau J . Recommendations for examining and interpreting funnel plot asymmetry in meta-analyses of randomised controlled trials. BMJ (Clinical Res ed) (2011) 343:d4002. doi: 10.1136/bmj.d4002 21784880

[19] Higgins JP Altman DG Gøtzsche PC Jüni P Moher D Oxman AD . The cochrane collaboration’s tool for assessing risk of bias in randomised trials. Bmj (2011) 343:d5928. doi: 10.1136/bmj.d5928 22008217 PMC3196245

[20] Viechtbauer W . Conducting meta-analyses in r with the metafor package. J Stat Software (2010) 36(3):1–48. doi: 10.18637/jss.v036.i03

[21] Planchard D Reinmuth N Orlov S Fischer JR Sugawara S Mandziuk S . ARCTIC: durvalumab with or without tremelimumab as third-line or later treatment of metastatic non-small-cell lung cancer. Ann Oncol (2020) 31(5):609–18. doi: 10.1016/j.annonc.2020.02.006 32201234

[22] Reck M Rodríguez-Abreu D Robinson AG Hui R Csőszi T Fülöp A . Updated analysis of KEYNOTE-024: pembrolizumab versus platinum-based chemotherapy for advanced non-Small-Cell lung cancer with PD-L1 tumor proportion score of 50% or greater. J Clin Oncol (2019) 37(7):537–46. doi: 10.1200/JCO.18.00149 30620668

[23] Reck M Wehler T Orlandi F Nogami N Barone C Moro-Sibilot D . Safety and patient-reported outcomes of atezolizumab plus chemotherapy with or without bevacizumab versus bevacizumab plus chemotherapy in non-small-cell lung cancer. J Clin Oncol (2020) 38(22):2530–42. doi: 10.1200/JCO.19.03158 PMC7392741 32459597

[24] Wu YL Lu S Cheng Y Zhou C Wang J Mok T . Nivolumab versus docetaxel in a predominantly Chinese patient population with previously treated advanced NSCLC: CheckMate 078 randomized phase III clinical trial. J Thorac Oncol (2019) 14(5):867–75. doi: 10.1016/j.jtho.2019.01.006 30659987

[25] Rizvi NA Cho BC Reinmuth N Lee KH Luft A Ahn MJ . Durvalumab with or without tremelimumab vs standard chemotherapy in first-line treatment of metastatic non-small cell lung cancer: the MYSTIC phase 3 randomized clinical trial. JAMA Oncol (2020) 6(5):661–74. doi: 10.1001/jamaoncol.2020.0237 PMC7146551 32271377

[26] Jotte R Cappuzzo F Vynnychenko I Stroyakovskiy D Rodríguez-Abreu D Hussein M . Atezolizumab in combination with carboplatin and nab-paclitaxel in advanced squamous NSCLC (IMpower131): results from a randomized phase III trial. J Thorac Oncol (2020) 15(8):1351–60. doi: 10.1016/j.jtho.2020.03.028 32302702

[27] Nishio M Barlesi F West H Ball S Bordoni R Cobo M . Atezolizumab plus chemotherapy for first-line treatment of nonsquamous NSCLC: results from the randomized phase 3 IMpower132 trial. J Thorac Oncol (2021) 16(4):653–64. doi: 10.1016/j.jtho.2020.11.025 33333328

[28] Yang Y Wang Z Fang J Yu Q Han B Cang S . Efficacy and safety of sintilimab plus pemetrexed and platinum as first-line treatment for locally advanced or metastatic nonsquamous NSCLC: a randomized, double-blind, phase 3 study (Oncology pRogram by InnovENT anti-PD-1-11). J Thorac Oncol (2020) 15(10):1636–46. doi: 10.1016/j.jtho.2020.07.014 32781263

[29] Felip E Altorki N Zhou C Csőszi T Vynnychenko I Goloborodko O . Adjuvant atezolizumab after adjuvant chemotherapy in resected stage IB-IIIA non-small-cell lung cancer (IMpower010): a randomised, multicentre, open-label, phase 3 trial. Lancet (2021) 398(10308):1344–57. doi: 10.1016/S0140-6736(21)02098-5 34555333

[30] Gandhi L Rodríguez-Abreu D Gadgeel S Esteban E Felip E De Angelis F . Pembrolizumab plus chemotherapy in metastatic non-Small-Cell lung cancer. N Engl J Med (2018) 378(22):2078–92. doi: 10.1056/NEJMoa1801005 29658856

[31] West H McCleod M Hussein M Morabito A Rittmeyer A Conter HJ . Atezolizumab in combination with carboplatin plus nab-paclitaxel chemotherapy compared with chemotherapy alone as first-line treatment for metastatic non-squamous non-small-cell lung cancer (IMpower130): a multicentre, randomised, open-label, phase 3 trial. Lancet Oncol (2019) 20(7):924–37. doi: 10.1016/S1470-2045(19)30167-6 31122901

[32] Paz-Ares L Ciuleanu TE Cobo M Schenker M Zurawski B Menezes J . First-line nivolumab plus ipilimumab combined with two cycles of chemotherapy in patients with non-small-cell lung cancer (CheckMate 9LA): an international, randomised, open-label, phase 3 trial. Lancet Oncol (2021) 22(2):198–211. doi: 10.1016/S1470-2045(20)30641-0 33476593

[33] Sezer A Kilickap S Gümüş M Bondarenko I Özgüroğlu M Gogishvili M . Cemiplimab monotherapy for first-line treatment of advanced non-small-cell lung cancer with PD-L1 of at least 50%: a multicentre, open-label, global, phase 3, randomised, controlled trial. Lancet (2021) 397(10274):592–604. doi: 10.1016/S0140-6736(21)00228-20 33581821

[34] Mok TSK Wu YL Kudaba I Kowalski DM Cho BC Turna HZ . Pembrolizumab versus chemotherapy for previously untreated, PD-L1-expressing, locally advanced or metastatic non-small-cell lung cancer (KEYNOTE-042): a randomised, open-label, controlled, phase 3 trial. Lancet (2019) 393(10183):1819–30. doi: 10.1016/S0140-6736(18)32409-7 30955977

[35] Wang Z Wu L Li B Cheng Y Li X Wang X . Toripalimab plus chemotherapy for patients with treatment-naive advanced non-Small-Cell lung cancer: a multicenter randomized phase III trial (CHOICE-01). J Clin Oncol (2023) 41(3):651–63. doi: 10.1200/JCO.22.00727 PMC9870236 36206498

[36] O’Brien M Paz-Ares L Marreaud S Dafni U Oselin K Havel L . Pembrolizumab versus placebo as adjuvant therapy for completely resected stage IB-IIIA non-small-cell lung cancer (PEARLS/KEYNOTE-091): an interim analysis of a randomised, triple-blind, phase 3 trial. Lancet Oncol (2022) 23(10):1274–86. doi: 10.1016/S1470-2045(22)00518-6 36108662

[37] Gogishvili M Melkadze T Makharadze T Giorgadze D Dvorkin M Penkov K . Cemiplimab plus chemotherapy versus chemotherapy alone in non-small cell lung cancer: a randomized, controlled, double-blind phase 3 trial. Nat Med (2022) 28(11):2374–80. doi: 10.1038/s41591-022-01977-y PMC9671806 36008722

[38] de Castro G Jr. Rizvi NA Schmid P Syrigos K Martin C Yamamoto N . NEPTUNE: phase 3 study of first-line durvalumab plus tremelimumab in patients with metastatic NSCLC. J Thorac Oncol (2023) 18(1):106–19. doi: 10.1016/j.jtho.2022.09.223 36240972

[39] Peters S Dziadziuszko R Morabito A Felip E Gadgeel SM Cheema P . Atezolizumab versus chemotherapy in advanced or metastatic NSCLC with high blood-based tumor mutational burden: primary analysis of BFAST cohort c randomized phase 3 trial. Nat Med (2022) 28(9):1831–9. doi: 10.1038/s41591-022-01933-w PMC9499854 35995953

[40] Reck M Luft A Szczesna A Havel L Kim SW Akerley W . Phase III randomized trial of ipilimumab plus etoposide and platinum versus placebo plus etoposide and platinum in extensive-stage small-cell lung cancer. J Clin Oncol (2016) 34(31):3740–8. doi: 10.1200/JCO.2016.67.6601 27458307

[41] Rudin CM Awad MM Navarro A Gottfried M Peters S Csőszi T . Pembrolizumab or placebo plus etoposide and platinum as first-line therapy for extensive-stage small-cell lung cancer: randomized, double-blind, phase III KEYNOTE-604 study. J Clin Oncol (2020) 38(21):2369–79. doi: 10.1200/JCO.20.00793 PMC7474472 32468956

[42] Goldman JW Dvorkin M Chen Y Reinmuth N Hotta K Trukhin D . Durvalumab, with or without tremelimumab, plus platinum-etoposide versus platinum-etoposide alone in first-line treatment of extensive-stage small-cell lung cancer (CASPIAN): updated results from a randomised, controlled, open-label, phase 3 trial. Lancet Oncol (2021) 22(1):51–65. doi: 10.1016/S1470-2045(20)30539-8 33285097

[43] Larkin J Minor D D’Angelo S Neyns B Smylie M Miller WH Jr. . Overall survival in patients with advanced melanoma who received nivolumab versus investigator’s choice chemotherapy in CheckMate 037: a randomized, controlled, open-label phase III trial. J Clin Oncol (2018) 36(4):383–90. doi: 10.1200/JCO.2016.71.8023 PMC6804912 28671856

[44] Ribas A Kefford R Marshall MA Punt CJ Haanen JB Marmol M . Phase III randomized clinical trial comparing tremelimumab with standard-of-care chemotherapy in patients with advanced melanoma. J Clin Oncol (2013) 31(5):616–22. doi: 10.1200/JCO.2012.44.6112 PMC4878048 23295794

[45] Gutzmer R Stroyakovskiy D Gogas H Robert C Lewis K Protsenko S . Atezolizumab, vemurafenib, and cobimetinib as first-line treatment for unresectable advanced BRAFV600 mutation-positive melanoma (IMspire150): primary analysis of the randomised, double-blind, placebo-controlled, phase 3 trial. Lancet (2020) 395(10240):1835–44. doi: 10.1016/S0140-6736(20)30934-X 32534646

[46] Weber JS D’Angelo SP Minor D Hodi FS Gutzmer R Neyns B . Nivolumab versus chemotherapy in patients with advanced melanoma who progressed after anti-CTLA-4 treatment (CheckMate 037): a randomised, controlled, open-label, phase 3 trial. Lancet Oncol (2015) 16(4):375–84. doi: 10.1016/S1470-2045(15)70076-8 25795410

[47] Atkins MB Lee SJ Chmielowski B Tarhini AA Cohen GI Truong TG . Combination dabrafenib and trametinib versus combination nivolumab and ipilimumab for patients with advanced BRAF-mutant melanoma: the DREAMseq trial-ECOG-ACRIN EA6134. J Clin Oncol (2023) 41(2):186–97. doi: 10.1200/JCO.22.01763 PMC9839305 36166727

[48] Long GV Luke JJ Khattak MA de la Cruz Merino L Del Vecchio M Rutkowski P . Pembrolizumab versus placebo as adjuvant therapy in resected stage IIB or IIC melanoma (KEYNOTE-716): distant metastasis-free survival results of a multicentre, double-blind, randomised, phase 3 trial. Lancet Oncol (2022) 23(11):1378–88. doi: 10.1016/S0140-6736(22)00562-1 36265502

[49] Bang YJ Ruiz EY Van Cutsem E Lee KW Wyrwicz L Schenker M . Randomised trial of avelumab versus physician’s choice of chemotherapy as third-line treatment of patients with advanced gastric or gastro-oesophageal junction cancer: primary analysis of JAVELIN gastric 300. Ann Oncol (2018) 29(10):2052–60. doi: 10.1093/annonc/mdy264 PMC6225815 30052729

[50] Moehler Markus . Phase III trial of avelumab maintenance after first-line induction chemotherapy versus continuation of chemotherapy in patients with gastric cancers: results from JAVELIN gastric 100. J Clin Oncol (2020) 39(9):966–77. doi: 10.1200/JCO.20.00892 PMC8078426 33197226

[51] Shitara K Van Cutsem E Bang YJ Fuchs C Wyrwicz L Lee KW . Efficacy and safety of pembrolizumab or pembrolizumab plus chemotherapy vs chemotherapy alone for patients with first-line, advanced gastric cancer: the KEYNOTE-062 phase 3 randomized clinical trial. JAMA Oncol (2020) 6(10):1571–80. doi: 10.1001/jamaoncol.2020.3370 PMC7489405 32880601

[52] Janjigian YY Shitara K Moehler M Garrido M Salman P Shen L . First-line nivolumab plus chemotherapy versus chemotherapy alone for advanced gastric, gastro-oesophageal junction, and oesophageal adenocarcinoma (CheckMate 649): a randomised, open-label, phase 3 trial. Lancet (2021) 398(10294):27–40. doi: 10.1016/S0140-6736(21)00797-2 34102137 PMC8436782

[53] Kang Yoon-Koo . Nivolumab plus chemotherapy versus placebo plus chemotherapy in patients with HER2-negative, untreated, unresectable advanced or recurrent gastric or gastro-oesophageal junction cancer (ATTRACTION-4): a randomised, multicentre, double-blind, placebo-controlled, phase 3 trial. (2021). doi: 10.1016/S1470-2045(21)00692-6 35030335

[54] Shitara K Özgüroğlu M Bang YJ Di Bartolomeo M Mandalà M Ryu MH . Pembrolizumab versus paclitaxel for previously treated, advanced gastric or gastro-oesophageal junction cancer (KEYNOTE-061): a randomised, open-label, controlled, phase 3 trial. Lancet (2018) 392(10142):123–33. doi: 10.1016/S0140-6736(18)31257-1 29880231

[55] Bajorin DF Witjes JA Gschwend JE Schenker M Valderrama BP Tomita Y . Adjuvant nivolumab versus placebo in muscle-invasive urothelial carcinoma. N Engl J Med (2021) 384(22):2102–14. doi: 10.1056/NEJMoa2034442 PMC8215888 34077643

[56] Bellmunt J Hussain M Gschwend JE Albers P Oudard S Castellano D . Adjuvant atezolizumab versus observation in muscle-invasive urothelial carcinoma (IMvigor010): a multicentre, open-label, randomised, phase 3 trial. Lancet Oncol (2021) 22(4):525–37. doi: 10.1016/S1470-2045(21)00004-8 PMC8495594 33721560

[57] Powles T van der Heijden MS Castellano D Galsky MD Loriot Y Petrylak DP . Durvalumab alone and durvalumab plus tremelimumab versus chemotherapy in previously untreated patients with unresectable, locally advanced or metastatic urothelial carcinoma (DANUBE): a randomised, open-label, multicentre, phase 3 trial. Lancet Oncol (2020) 21(12):1574–88. doi: 10.1016/S1470-2045(20)30541-6 32971005

[58] Powles T Csőszi T Özgüroğlu M Matsubara N Géczi L Cheng SY . Pembrolizumab alone or combined with chemotherapy versus chemotherapy as first-line therapy for advanced urothelial carcinoma (KEYNOTE-361): a randomised, open-label, phase 3 trial. Lancet Oncol (2021) 22(7):931–45. doi: 10.1016/S1470-2045(21)00152-2 34051178

[59] Motzer RJ Tannir NM McDermott DF Arén Frontera O Melichar B Choueiri TK . Nivolumab plus ipilimumab versus sunitinib in advanced renal-cell carcinoma. N Engl J Med (2018) 378(14):1277–90. doi: 10.1056/NEJMoa1712126 PMC5972549 29562145

[60] Choueiri TK Powles T Burotto M Escudier B Bourlon MT Zurawski B . Nivolumab plus cabozantinib versus sunitinib for advanced renal-cell carcinoma. N Engl J Med (2021) 384(9):829–41. doi: 10.1056/NEJMoa2026982 PMC8436591 33657295

[61] Powles T Plimack ER Soulières D Waddell T Stus V Gafanov R . Pembrolizumab plus axitinib versus sunitinib monotherapy as first-line treatment of advanced renal cell carcinoma (KEYNOTE-426): extended follow-up from a randomised, open-label, phase 3 trial. Lancet Oncol (2020) 21(12):1563–73. doi: 10.1016/S1470-2045(20)30436-8 33284113

[62] Pal SK Uzzo R Karam JA Master VA Donskov F Suarez C . Adjuvant atezolizumab versus placebo for patients with renal cell carcinoma at increased risk of recurrence following resection (IMmotion010): a multicentre, randomised, double-blind, phase 3 trial. Lancet (2022) 400(10358):1103–16. doi: 10.1016/S0140-6736(22)01658-0 36099926

[63] Mittendorf EA Zhang H Barrios CH Saji S Jung KH Hegg R . Neoadjuvant atezolizumab in combination with sequential nab-paclitaxel and anthracycline-based chemotherapy versus placebo and chemotherapy in patients with early-stage triple-negative breast cancer (IMpassion031): a randomised, double-blind, phase 3 trial. Lancet (2020) 396(10257):1090–100. doi: 10.1016/S0140-6736(20)31953-X 32966830

[64] Burtness B Harrington KJ Greil R Soulières D Tahara M de Castro G Jr. . Pembrolizumab alone or with chemotherapy versus cetuximab with chemotherapy for recurrent or metastatic squamous cell carcinoma of the head and neck (KEYNOTE-048): a randomised, open-label, phase 3 study. Lancet (2019) 394(10212):1915–28. doi: 10.1016/S0140-6736(19)32591-7 31679945

[65] Cohen EEW Soulières D Le Tourneau C Dinis J Licitra L Ahn MJ . Pembrolizumab versus methotrexate, docetaxel, or cetuximab for recurrent or metastatic head-and-neck squamous cell carcinoma (KEYNOTE-040): a randomised, open-label, phase 3 study. Lancet (2019) 393(10167):156–67. doi: 10.1016/S0140-6736(18)31999-8 30509740

[66] Lee NY Ferris RL Psyrri A Haddad RI Tahara M Bourhis J . Avelumab plus standard-of-care chemoradiotherapy versus chemoradiotherapy alone in patients with locally advanced squamous cell carcinoma of the head and neck: a randomised, double-blind, placebo-controlled, multicentre, phase 3 trial. Lancet Oncol (2021) 22(4):450–62. doi: 10.1016/S1470-2045(20)30737-3 33794205

[67] Kwon ED Drake CG Scher HI Fizazi K Bossi A van den Eertwegh AJ . Ipilimumab versus placebo after radiotherapy in patients with metastatic castration-resistant prostate cancer that had progressed after docetaxel chemotherapy (CA184-043): a multicentre, randomised, double-blind, phase 3 trial. Lancet Oncol (2014) 15(7):700–12. doi: 10.1016/S1470-2045(14)70189-5 PMC4418935 24831977

[68] Ren Z Xu J Bai Y Xu A Cang S Du C . Sintilimab plus a bevacizumab biosimilar (IBI305) versus sorafenib in unresectable hepatocellular carcinoma (ORIENT-32): a randomised, open-label, phase 2–3 study. Lancet Oncol (2021) 22(7):977–90. doi: 10.1016/S1470-2045(21)00252-7 34143971

[69] Cheng AL Qin S Ikeda M Galle PR Ducreux M Kim TY . Updated efficacy and safety data from IMbrave150: atezolizumab plus bevacizumab vs. sorafenib for unresectable hepatocellular carcinoma. J Hepatol (2022) 76(4):862–73. doi: 10.1016/j.jhep.2021.11.030 34902530

[70] Kelley RK Rimassa L Cheng AL Kaseb A Qin S Zhu AX . Cabozantinib plus atezolizumab versus sorafenib for advanced hepatocellular carcinoma (COSMIC-312): a multicentre, open-label, randomised, phase 3 trial. Lancet Oncol (2022) 23(8):995–1008. doi: 10.1016/S1470-2045(22)00326-6 35798016

[71] Huang J Xu J Chen Y Zhuang W Zhang Y Chen Z . Camrelizumab versus investigator’s choice of chemotherapy as second-line therapy for advanced or metastatic oesophageal squamous cell carcinoma (ESCORT): a multicentre, randomised, open-label, phase 3 study. Lancet Oncol (2020) 21(6):832–42. doi: 10.1016/S1470-2045(20)30110-8 32416073

[72] Sun JM Shen L Shah MA Enzinger P Adenis A Doi T . Pembrolizumab plus chemotherapy versus chemotherapy alone for first-line treatment of advanced oesophageal cancer (KEYNOTE-590): a randomised, placebo-controlled, phase 3 study. Lancet (2021) 398(10302):759–71. doi: 10.1016/S0140-6736(21)01234-4 34454674

[73] Moore KN Bookman M Sehouli J Miller A Anderson C Scambia G . Atezolizumab, bevacizumab, and chemotherapy for newly diagnosed stage III or IV ovarian cancer: placebo-controlled randomized phase III trial (IMagyn050/GOG 3015/ENGOT-OV39). J Clin Oncol (2021) 39(17):1842–55. doi: 10.1200/JCO.21.00306 PMC8189598 33891472

[74] Pujade-Lauraine E Fujiwara K Ledermann JA Oza AM Kristeleit R Ray-Coquard IL . Avelumab alone or in combination with chemotherapy versus chemotherapy alone in platinum-resistant or platinum-refractory ovarian cancer (JAVELIN ovarian 200): an open-label, three-arm, randomised, phase 3 study. Lancet Oncol (2021) 22(7):1034–46. doi: 10.1016/S1470-2045(21)00216-3 34143970

[75] Monk BJ Colombo N Oza AM Fujiwara K Birrer MJ Randall L . Chemotherapy with or without avelumab followed by avelumab maintenance versus chemotherapy alone in patients with previously untreated epithelial ovarian cancer (JAVELIN ovarian 100): an open-label, randomised, phase 3 trial. Lancet Oncol (2021) 22(9):1275–89. doi: 10.1016/S1470-2045(21)00342-9 34363762

[76] Eng C Kim TW Bendell J Argilés G Tebbutt NC Di Bartolomeo M . Atezolizumab with or without cobimetinib versus regorafenib in previously treated metastatic colorectal cancer (IMblaze370): a multicentre, open-label, phase 3, randomised, controlled trial. Lancet Oncol (2019) 20(6):849–61. doi: 10.1016/S1470-2045(19)30027-0 31003911

[77] Reardon DA Brandes AA Omuro A Mulholland P Lim M Wick A . Effect of nivolumab vs bevacizumab in patients with recurrent glioblastoma: the CheckMate 143 phase 3 randomized clinical trial. JAMA Oncol (2020) 6(7):1003–10. doi: 10.1001/jamaoncol.2020.1024 PMC7243167 32437507

[78] Baas P Scherpereel A Nowak AK Fujimoto N Peters S Tsao AS . First-line nivolumab plus ipilimumab in unresectable malignant pleural mesothelioma (CheckMate 743): a multicentre, randomised, open-label, phase 3 trial. Lancet (2021) 397(10272):375–86. doi: 10.1016/S0140-6736(20)32714-8 33485464

[79] Fennell DA Ewings S Ottensmeier C Califano R Hanna GG Hill K . Nivolumab versus placebo in patients with relapsed malignant mesothelioma (CONFIRM): a multicentre, double-blind, randomised, phase 3 trial. Lancet Oncol (2021) 22(11):1530–40. doi: 10.1016/S1470-2045(21)00471-X PMC8560642 34656227

[80] Moehler M Dvorkin M Boku N Özgüroğlu M Ryu MH Muntean AS . Phase III trial of avelumab maintenance after first-line induction chemotherapy versus continuation of chemotherapy in patients with gastric cancers: results from JAVELIN gastric 100. J Clin Oncol (2021) 39(9):966–77. doi: 10.1200/JCO.20.00892 PMC8078426 33197226

[81] Kang YK Chen LT Ryu MH Oh DY Oh SC Chung HC . Nivolumab plus chemotherapy versus placebo plus chemotherapy in patients with HER2-negative, untreated, unresectable advanced or recurrent gastric or gastro-oesophageal junction cancer (ATTRACTION-4): a randomised, multicentre, double-blind, placebo-controlled, phase 3 trial. Lancet Oncol (2022) 23(2):234–47. doi: 10.1016/S1470-2045(21)00692-6 35030335

[82] George J Bajaj D Sankaramangalam K Yoo JW Joshi NS Gettinger S . Incidence of pancreatitis with the use of immune checkpoint inhibitors (ICI) in advanced cancers: a systematic review and meta-analysis. Pancreatology (2019) 19(4):587–94. doi: 10.1016/j.pan.2019.04.015 31076344

[83] Friedman CF Clark V Raikhel AV Barz T Shoushtari AN Momtaz P . Thinking critically about classifying adverse events: incidence of pancreatitis in patients treated with nivolumab + ipilimumab. J Natl Cancer Inst (2017) 109(4):djw260. doi: 10.1093/jnci/djw260 28040701 PMC5441295

[84] Abu-Sbeih H Tang T Lu Y Thirumurthi S Altan M Jazaeri AA . Clinical characteristics and outcomes of immune checkpoint inhibitor-induced pancreatic injury. J immunother cancer (2019) 7(1):31. doi: 10.1186/s40425-019-0502-7 30728076 PMC6364483

[85] Brahmer JR Lacchetti C Schneider BJ Atkins MB Brassil KJ Caterino JM . Management of immune-related adverse events in patients treated with immune checkpoint inhibitor therapy: American society of clinical oncology clinical practice guideline. J Clin Oncol (2018) 36(17):1714–68. doi: 10.1200/JCO.2017.77.6385 PMC6481621 29442540

[86] Ikeuchi K Okuma Y Tabata T . Immune-related pancreatitis secondary to nivolumab in a patient with recurrent lung adenocarcinoma: a case report. Lung Cancer (Amsterdam Netherlands) (2016) 99:148–50. doi: 10.1016/j.lungcan.2016.07.001 27565931

[87] Hofmann L Forschner A Loquai C Goldinger SM Zimmer L Ugurel S . Cutaneous, gastrointestinal, hepatic, endocrine, and renal side-effects of anti-PD-1 therapy. Eur J Cancer (Oxford England: 1990) (2016) 60:190–209. doi: 10.1016/j.ejca.2016.02.025 27085692

[88] Zhang Y Fang Y Wu J Huang G Bin J Liao Y . Pancreatic adverse events associated with immune checkpoint inhibitors: a Large-scale pharmacovigilance analysis. Front Pharmacol (2022) 13:817662. doi: 10.3389/fphar.2022.817662 35431928 PMC9012537

[89] Marchand L Disse E Dalle S Reffet S Vouillarmet J Fabien N . The multifaceted nature of diabetes mellitus induced by checkpoint inhibitors. Acta diabetologica (2019) 56(12):1239–45. doi: 10.1007/s00592-019-01402-w 31423559

[90] Hsu C Marshall JL He AR . Workup and management of immune-mediated hepatobiliary pancreatic toxicities that develop during immune checkpoint inhibitor treatment. oncologist (2020) 25(2):105–11. doi: 10.1634/theoncologist.2018-0162 PMC7011649 32043797

[91] Passat T Touchefeu Y Gervois N Jarry A Bossard C Bennouna J . Physiopathological mechanisms of immune-related adverse events induced by anti-CTLA-4, anti-PD-1 and anti-PD-L1 antibodies in cancer treatment. Bull du cancer (2018) 105(11):1033–41. doi: 10.1016/j.bulcan.2018.07.005 30244981

[92] Yoneda S Imagawa A Hosokawa Y Baden MY Kimura T Uno S . T-Lymphocyte infiltration to islets in the pancreas of a patient who developed type 1 diabetes after administration of immune checkpoint inhibitors. Diabetes Care (2019) 42(7):e116–e8. doi: 10.2337/dc18-2518 31076419

[93] Weber J Kähler K Hauschild A . Management of immune-related adverse events and kinetics of response with ipilimumab. J Clin Oncol (2012) 30(21):2691–7. doi: 10.1200/JCO.2012.41.6750 22614989

[94] Darvin P Toor SM Sasidharan Nair V Elkord E . Immune checkpoint inhibitors: recent progress and potential biomarkers. Exp Mol Med (2018) 50(12):1–11. doi: 10.1038/s12276-018-0191-1 PMC6292890 30546008

[95] Nagpal S Sharma A Chari S . Autoimmune pancreatitis. Am J gastroenterol (2018) 113(9):1301. doi: 10.1038/s41395-018-0146-0 29910463

[96] Arbour KC Mezquita L Long N Rizvi H Auclin E Ni A . Impact of baseline steroids on efficacy of programmed cell death-1 and programmed death-ligand 1 blockade in patients with non-Small-Cell lung cancer. J Clin Oncol (2018) 36(28):2872–8. doi: 10.1200/JCO.2018.79.0006 30125216

[97] Pollack MH Betof A Dearden H Rapazzo K Valentine I Brohl AS . Safety of resuming anti-PD-1 in patients with immune-related adverse events (irAEs) during combined anti-CTLA-4 and anti-PD1 in metastatic melanoma. Ann Oncol (2018) 29(1):250–5. doi: 10.1093/annonc/mdx642 PMC5834131 29045547

[98] Spector D Perry Z Shah S Kim JJ Tarnoff ME Shikora SA . Roux-en-Y gastric bypass: hyperamylasemia is associated with small bowel obstruction. Surg Obes related Dis (2015) 11(1):38–43. doi: 10.1016/j.soard.2014.04.030 25264325

[99] Coté GA Gottstein JH Daud A Blei AT . The role of etiology in the hyperamylasemia of acute liver failure. Am J Gastroenterol (2009) 104(3):592–7. doi: 10.1038/ajg.2008.84 PMC3641762 19223884

[100] Ismail OZ Bhayana V . Lipase or amylase for the diagnosis of acute pancreatitis? Clin Biochem (2017) 50(18):1275–80. doi: 10.1016/j.clinbiochem.2017.07.003 28720341

[101] Michot JM Ragou P Carbonnel F Champiat S Voisin AL Mateus C . Significance of immune-related lipase increase induced by antiprogrammed death-1 or death ligand-1 antibodies: a brief communication. J immunother (Hagerstown Md: 1997) (2018) 41(2):84–5. doi: 10.1097/CJI.0000000000000202 29252914

[102] Larkin J Chiarion-Sileni V Gonzalez R Grob JJ Cowey CL Lao CD . Combined nivolumab and ipilimumab or monotherapy in untreated melanoma. N Engl J Med (2015) 373(1):23–34. doi: 10.1056/NEJMoa1504030 26027431 PMC5698905

[103] Wolchok JD Chiarion-Sileni V Gonzalez R Rutkowski P Grob JJ Cowey CL . Overall survival with combined nivolumab and ipilimumab in advanced melanoma. N Engl J Med (2017) 377(14):1345–56. doi: 10.1056/NEJMoa1709684 PMC5706778 28889792

[104] Page MJ Sterne JAC Higgins JPT Egger M . Investigating and dealing with publication bias and other reporting biases in meta-analyses of health research: a review. Res synthesis Methods (2021) 12(2):248–59. doi: 10.1002/jrsm.1468 33166064

