# Supplementary material for: Pancreatic adverse events of immune checkpoint inhibitors therapy for solid cancer patients: a systematic review and meta-analysis
Source: Front Immunol. 2023 Jun 9;14:1166299. doi: 10.3389/fimmu.2023.1166299 (PMC10289552; doi:10.3389/fimmu.2023.1166299)
Supplement: Supplementary file 1 [file Table_1.docx]

| **Supplementary Table 1**. **Search strategies for systematic review. Research was conducted up to March 15, 2023, in PubMed, Embase and Cochrane Library.** | | |
| --- | --- | --- |
| Database | Keywords | Records |
| PubMed |  |  |
| **#1** | (((((((((Neoplasms) OR Neoplasia) OR Neoplasias) OR Neoplasm) OR Tumors) OR Tumor) OR Malignancy) OR  Malignancies) OR Cancer) OR Cancers | **5645797** |
| **#2** | (((((((((((((((Pembrolizumab)) OR (Nivolumab)) OR (Tislelizumab)) OR (Sintilimab)) OR (Camrelizumab)) OR (Toripalimab)) OR (atezolizumab)) OR (avelumab)) OR (Durvalumab)) OR (cemiplimab)) OR (tremelimumab)) OR (Ipilimumab)) OR (anti-PD-1)) OR (anti-PD-L1)) OR  (anti-CTLA-4) | **24741** |
| **#3** | ((((((randomized controlled trail) OR controlled clinicaltrail) OR  randomized) OR randomly) OR trial)) | **2641333** |
| **#4** | #1 AND #2 AND #3 | **8669** |
| **Embase** |  |  |
| **#1** | acral AND ('tumor'/exp OR 'tumor' OR 'tumor'/exp OR tumor) OR 'neoplasms'/exp OR 'neoplasms' OR 'neoplasms'/exp OR neoplasms OR (acral AND ('tumour'/exp OR 'tumour' OR 'tumour'/exp OR tumour)) OR (('neoplasms'/exp OR 'neoplasms' OR 'neoplasms'/exp OR neoplasms) AND by AND histologic AND type) OR (('neoplasms,'/exp OR neoplasms,) AND cystic, AND mucinous, AND serous) OR (('neoplasms,'/exp OR neoplasms,) AND embryonal AND mixed) OR (('neoplasms,'/exp OR neoplasms,) AND ('germ'/exp OR 'germ' OR 'germ'/exp OR germ) AND ('cell'/exp OR 'cell' OR 'cell'/exp OR cell) AND embryonal;) OR (('neoplasms,'/exp OR neoplasms,) AND glandular AND epithelial;) OR (('neoplasms,'/exp OR neoplasms,) AND 'hormone dependent;') OR (('neoplasms,'/exp OR neoplasms,) AND 'post traumatic') OR (neoplastic AND ('disease'/exp OR 'disease' OR 'disease'/exp OR disease)) OR 'tumor'/exp OR 'tumor' OR 'tumor'/exp OR tumor OR  'tumour'/exp OR 'tumour' OR 'tumour'/exp OR tumour | **6759352** |
| **#2** | 'ipilimumab'/exp OR 'ipilimumab' OR 'ipilimumab'/exp OR ipilimumab OR 'tremelimumab'/exp OR 'tremelimumab' OR 'tremelimumab'/exp OR tremelimumab OR 'nivolumab'/exp OR 'nivolumab' OR 'nivolumab'/exp OR nivolumab OR 'pembrolizumab'/exp OR 'pembrolizumab' OR 'pembrolizumab'/exp OR pembrolizumab OR 'avelumab'/exp OR 'avelumab' OR 'avelumab'/exp OR avelumab OR 'atezolizumab'/exp OR 'atezolizumab' OR  'atezolizumab'/exp OR atezolizumab OR 'durvalumab'/exp | **66535** |

|  | OR 'durvalumab' OR 'durvalumab'/exp OR durvalumab OR 'tislelizumab'/exp OR 'tislelizumab' OR 'sintilimab'/exp OR 'sintilimab' OR 'camrelizumab'/exp OR camrelizumab OR 'camrelizumab'/exp OR 'camrelizumab' OR 'toripalimab'/exp OR 'toripalimab' OR 'cemiplimab'/exp OR  'cemiplimab' |  |
| --- | --- | --- |
| **#3** | randomized AND controlled AND ('trial'/exp OR 'trial' OR 'trial'/exp OR trial) OR (controlled AND trial, AND randomized;) OR 'randomized controlled trial'/exp OR 'randomized controlled trial' OR (pragmatic AND ('clinical'/exp OR 'clinical' OR 'clinical'/exp OR clinical) AND trials) OR (randomised AND controlled AND ('study'/exp OR 'study' OR 'study'/exp OR study)) OR (randomised AND controlled AND ('trial'/exp OR 'trial' OR 'trial'/exp OR trial)) OR (randomized AND controlled AND study;) OR (trial,  AND randomized AND controlled) | **1326990** |
| **#4** | #1 AND #2 AND #3 | **8315** |
| **Cochrane Library** |  |  |
| **#1** | (Pembrolizumab OR Nivolumab OR Tislelizumab OR Sintilimab OR Camrelizumab OR Toripalimab OR atezolizumab OR avelumab OR Durvalumab OR cemiplimab OR tremelimumab OR Ipilimumab OR anti-PD-1 OR anti-PD-L1 OR anti-CTLA-4) AND (' carcinoma ' OR ' cancer' OR ' tumor ') | **55895** |
| **#2** | randomized controlled trail | **8890** |
