# Supplementary material for: Pancreatic adverse events of immune checkpoint inhibitors therapy for solid cancer patients: a systematic review and meta-analysis
Source: Front Immunol. 2023 Jun 9;14:1166299. doi: 10.3389/fimmu.2023.1166299 (PMC10289552; doi:10.3389/fimmu.2023.1166299)
Supplement: Supplementary file 2 [file Table_2.docx]

| Supplementary Table 2. Risk of bias of each randomized controlled trial included for comparison. | | | | | | | |
| --- | --- | --- | --- | --- | --- | --- | --- |
| Study | Randomization | Allocation  concealment | Blinding of  participants  and staff | Blinding of  outcome  assessors | Incomplete  outcome data | Selective  outcome  reporting | Other  sources of  bias |
| D. Planchard  (2020) | **Low** | **Low** | **High** | **Low** | **Low** | **Low** | **Low** |
| Martin Reck  （2019） | **Low** | **Low** | **High** | **High** | **Low** | **Low** | **Low** |
| Martin Reck  （2019） | **Low** | **Low** | **High** | **High** | **Low** | **Low** | **Low** |
| Yi-Long Wu  (2019) | **Low** | **Low** | **Low** | **Low** | **Low** | **Low** | **Low** |
| Naiyer  A. Rizvi  (2020) | **Low** | **Low** | **High** | **Low** | **Low** | **Low** | **Low** |
| Robert Jotte  (2020) | **Low** | **Low** | **High** | **High** | **Low** | **Low** | **Low** |
| Makoto Nishio  (2021) | **Low** | **Low** | **High** | **High** | **Low** | **Low** | **Low** |
| Yunpeng Yang  (2020) | **Low** | **Low** | **Low** | **High** | **Low** | **Low** | **Low** |
| Enriqueta Felip  (2021) | **Low** | **Low** | **High** | **High** | **Low** | **Low** | **Low** |
| L. Gandhi  (2018) | **Low** | **Low** | **Low** | **High** | **Low** | **Low** | **Low** |
| Howard West  (2019) | **Low** | **Low** | **High** | **High** | **Low** | **Low** | **Low** |
| Luis Paz-Ares  (2021) | **Low** | **Low** | **High** | **High** | **Low** | **Low** | **Low** |
| Ahmet Sezer  (2021) | **Low** | **Low** | **High** | **High** | **Low** | **Low** | **Low** |
| Tony S K Mok  (2019) | **Low** | **Low** | **High** | **High** | **Low** | **Low** | **Low** |
| Martin Reck  (2016) | **Low** | **Low** | **Low** | **High** | **Low** | **Low** | **Low** |
| CharlesM.Rudin  (2020) | **Low** | **Low** | **Low** | **High** | **Low** | **Low** | **Low** |
| Jonathan  W Goldman  (2021) | **Low** | **Low** | **High** | **High** | **Low** | **Low** | **Low** |
| James Larkin  (2018) | **Low** | **Low** | **High** | **High** | **Low** | **Low** | **Low** |
| Antoni Ribas  (2013) | **Low** | **Low** | **High** | **High** | **Low** | **Low** | **Low** |
| Ralf Gutzmer  (2020) | **Low** | **Low** | **Low** | **High** | **Low** | **Low** | **Low** |
| Jeffff rey S Webe  (2015) | **Low** | **Low** | **High** | **High** | **Low** | **Low** | **Low** |
| Y.-J. Bang  (2018) | **Low** | **Low** | **High** | **High** | **Low** | **Low** | **Low** |
| Markus Moehler  (2020) | **Low** | **Low** | **High** | **High** | **Low** | **Low** | **Low** |
| Kohei Shitara  (2020) | **Low** | **Low** | **Low** | **High** | **Low** | **Low** | **Low** |
| Yelena Y Janjigian  (2021) | **Low** | **Low** | **High** | **High** | **Low** | **Low** | **Low** |
| Yoon-Koo Kang  (2021) | **Low** | **Low** | **Low** | **High** | **Low** | **Low** | **Low** |
| Kohei Shitara（2018） | **Low** | **Low** | **High** | **High** | **Low** | **Low** | **Low** |
| D.F. Bajorin  （2021） | **Low** | **Low** | **Low** | **High** | **Low** | **Low** | **Low** |
| Joaquim Bellmunt  (2021) | **Low** | **Low** | **High** | **High** | **Low** | **Low** | **Low** |
| Thomas Powles  (2020) | **Low** | **Low** | **High** | **High** | **Low** | **Low** | **Low** |
| Thomas Powles  (2021) | **Low** | **Low** | **High** | **High** | **Low** | **Low** | **Low** |
| R.J. Motzer  (2018) | **Low** | **Low** | **High** | **High** | **Low** | **Low** | **Low** |
| T.K.  Choueiri  (2021) | **Low** | **Low** | **High** | **High** | **Low** | **Low** | **Low** |
| Thomas Powles  (2020) | **Low** | **Low** | **High** | **High** | **Low** | **Low** | **Low** |
| Elizabeth A Mittendorf (2020) | **Low** | **Low** | **Low** | **High** | **Low** | **Low** | **Low** |
| Barbara Burtness  (2019) | **Low** | **Low** | **High** | **Lowl** | **Low** | **Low** | **Low** |
| Ezra E W Cohen  (2019) | **Low** | **Low** | **High** | **High** | **Low** | **Low** | **Low** |
| Nancy Y Lee  (2021) | **Low** | **Low** | **Low** | **High** | **Low** | **Low** | **Low** |
| Eugene D Kwon  (2014) | **Low** | **Low** | **Low** | **High** | **Low** | **Low** | **Low** |
| Zhenggang Ren  (2021) | **Low** | **Low** | **High** | **High** | **Low** | **Low** | **Low** |
| Jing Huang  (2020) | **Low** | **Low** | **High** | **High** | **Low** | **Low** | **Low** |
| Jong-Mu Sun  (2021) | **Low** | **Low** | **Low** | **High** | **Low** | **Low** | **Low** |
| Kathlen N.Moore  (2021) | **Low** | **Low** | **Low** | **High** | **Low** | **Low** | **Low** |
| Eric Pujade-Lauraine  (2021) | **Low** | **Low** | **High** | **High** | **Low** | **Low** | **Low** |
| Bradley J  Monk  (2021) | **Low** | **Low** | **High** | **High** | **Low** | **Low** | **Low** |
| Cathy Eng  (2019) | **Low** | **Low** | **High** | **High** | **Low** | **Low** | **Low** |
| David A. Reardon  (2020) | **Low** | **Low** | **High** | **High** | **Low** | **Low** | **Low** |
| Paul Baas  (2021) | **Low** | **Low** | **High** | **High** | **Low** | **Low** | **Low** |
| Dean A Fennel  (2021) | **Low** | **Low** | **Low** | **High** | **Low** | **Low** | **Low** |
| Z. Wang  (2023) | **Low** | **Low** | **Low** | **High** | **Low** | **Low** | **Low** |
| M. O'Brien  (2022) | **Low** | **Low** | **Low** | **Low** | **Low** | **Low** | **Low** |
| M. Gogishvili  (2022) | **Low** | **Low** | **Low** | **High** | **Low** | **Low** | **Low** |
| G. de Castro  (2023) | **Low** | **Low** | **High** | **High** | **Low** | **Low** | **Low** |
| S. Peters  (2022) | **Low** | **Low** | **High** | **High** | **Low** | **Low** | **Low** |
| M. B. Atkins  (2023) | **Low** | **Low** | **High** | **High** | **Low** | **Low** | **Low** |
| G. V. Long  (2022) | **Low** | **Low** | **Low** | **High** | **Low** | **Low** | **Low** |
| S. K. Pal  (2022) | **Low** | **Low** | **Low** | **High** | **Low** | **Low** | **Low** |
| A. L. Cheng  (2022) | **Low** | **Low** | **High** | **High** | **Low** | **Low** | **Low** |
| R. K. Kelley  (2022) | **Low** | **Low** | **High** | **High** | **Low** | **Low** | **Low** |

Low indicates no risk, high indicates high risk and unclear indicates unknown risk.
