# Supplementary material for: Pancreatic adverse events of immune checkpoint inhibitors therapy for solid cancer patients: a systematic review and meta-analysis
Source: Front Immunol. 2023 Jun 9;14:1166299. doi: 10.3389/fimmu.2023.1166299 (PMC10289552; doi:10.3389/fimmu.2023.1166299)
Supplement: Supplementary file 3 [file Table_3.docx]

| Supplementary Table 3. Incidences of ICI therapy-associated pancreatitis in randomized controlled trials. | | | | | | |
| --- | --- | --- | --- | --- | --- | --- |
| Variables | **Pancreatitis** | | | | | |
|  | **Grade 1-5** | | | **Grade 3-5** | | |
|  | **n/N** | **Incidence (%)** | **95%CI** | **n/N** | **Incidence**  **(%)** | **95%CI** |
| Combination type |  | | | | | |
| Single ICI therapy | 26/5678 | 0.70 | 0.51-0.97 | 12/4935 | 0.58 | 0.38-0.90 |
| ICI+ Chem/Targeted | 64/8526 | 0.96 | 0.71-1.29 | 30/7483 | 0.65 | 0.48-0.89 |
| Dual ICI therapy | 20/1973 | 1.10 | 0.71-1.71 | 12/1973 | 0.94 | 0.57-1.53 |
| Cancer type |  | | | | | |
| NSCLC | 40/6465 | 0.87 | 0.66-1.15 | 16/5672 | 0.57 | 0.38-0.87 |
| SCLC | 4/754 | 0.56 | 0.21-1.47 | 3/754 | 0.40 | 0.13-1.23 |
| Melanoma | 14/1439 | 1.11 | 0.60-2.04 | 6/1171 | 0.78 | 0.39-1.55 |
| GEJC | 2/798 | 0.52 | 0.14-1.87 | 0/294 | 0.17 | 0.01-2.65 |
| UC | 12/1726 | 0.77 | 0.41-1.43 | 8/1726 | 0.71 | 0.39-1.29 |
| RCC | 6/819 | 0.71 | 0.18-2.28 | 4/819 | 0.56 | 0.08-3.60 |
| BC | 0/164 | 0.30 | 0.02-4.65 | 0/164 | 0.30 | 0.02-4.65 |
| HNSCC | 3/576 | 0.54 | 0.18-1.67 | 1/576 | 0.40 | 0.10-1.60 |
| PC | - | - | - | - | - | - |
| HCC | 14/758 | 1.79 | 0.56-5.58 | 7/758 | 0.96 | 0.46-2.00 |
| ESO | 3/598 | 0.50 | 0.16-1.55 | 1/598 | 0.44 | 0.11-1.75 |
| OC | 6/1299 | 0.44 | 0.10-2.00 | 5/1299 | 0.40 | 0.11-1.44 |
| CRC | 3/269 | 1.12 | 0.36-3.40 | 3/269 | 1.12 | 0.36-3.40 |
| Glioblastoma | - | - | - | - | - | - |
| Mesothelioma | 3/521 | 0.59 | 0.19-1.80 | 0/300 | 0.17 | 0.01-2.60 |

ICI, immune checkpoint inhibitor; n/N refers to the number of events (n) observed for the outcome regarding the overall number of patients (N) in patients treated with immune checkpoint inhibitor therapy; CI, confidence interval. Chem, chemotherapy; Targeted, targeted therapy;NSCLC, non-small cell lung cancer; SCLC, small cell lung cancer; GEJC, gastroesophageal junction cancer; UC, urothelial carcinoma; RCC, renal cell carcinoma; BC, breast cancer; HNSCC, head and neck squamous cell carcinoma; PC, prostate cancer; HCC, hepatocellular carcinoma; ESO, esophageal carcinoma; OC, ovarian cancer; CRC, colorectal cancer.
