# Supplementary material for: Pancreatic adverse events of immune checkpoint inhibitors therapy for solid cancer patients: a systematic review and meta-analysis
Source: Front Immunol. 2023 Jun 9;14:1166299. doi: 10.3389/fimmu.2023.1166299 (PMC10289552; doi:10.3389/fimmu.2023.1166299)
Supplement: Supplementary file 4 [file Table_4.docx]

| Supplementary Table 4. Incidences of ICI therapy-associated amylase elevation in randomized controlled trials. | | | | | | |
| --- | --- | --- | --- | --- | --- | --- |
| Variables | **Amylase Elevation** | | | | | |
|  | **Grade 1-5** | | | **Grade 3-5** | | |
|  | **n/N** | **Incidence (%)** | **95%CI** | **n/N** | **Incidence**  **(%)** | **95%CI** |
| Combination type |  | | | | | |
| Single ICI therapy | 94/5122 | 1.66 | 1.04-2.63 | 32/5424 | 0.78 | 0.49-1.25 |
| ICI+ Chem/Targeted | 216/4699 | 3.78 | 2.14-6.60 | 74/4668 | 1.57 | 0.89-2.74 |
| Dual ICI therapy | 78/2412 | 3.01 | 1.29-5.98 | 37/2412 | 1.79 | 0.91-3.49 |
| Cancer type |  | | | | | |
| NSCLC | 88/4079 | 1.94 | 1.08-3.45 | 25/4079 | 0.85 | 0.48-1.50 |
| SCLC | 18/1009 | 1.59 | 0.34-7.18 | 7/1009 | 0.77 | 0.14-3.96 |
| Melanoma | 62/843 | 5.62 | 0.68-34.02 | 31/843 | 2.75 | 0.30-21.20 |
| GEJC | 12/602 | 1.33 | 0.09-17.55 | 2/602 | 0.56 | 0.09-3.32 |
| UC | 59/1426 | 3.47 | 1.51-7.77 | 38/2077 | 1.86 | 0.94-3.65 |
| RCC | 51/710 | 4.21 | 0.28-40.83 | 11/710 | 1.08 | 0.09-11.24 |
| BC | - | - | - | - | - | - |
| HNSCC | 21/594 | 1.85 | 0.14-20.65 | 7/594 | 1.11 | 0.30-4.02 |
| PC | 2/393 | 0.51 | 0.13-2.01 | 2/393 | 0.51 | 0.13-2.01 |
| HCC | 26/809 | 1.91 | 0.18-17.10 | 3/429 | 0.70 | 0.14-2.03 |
| ESO | - | - | - | - | - | - |
| OC | 21/1026 | 2.08 | 1.36-3.16 | 5/1026 | 0.66 | 0.31-1.42 |
| CRC | 8/269 | 3.02 | 1.52-5.93 | 3/269 | 1.66 | 0.62-4.34 |
| Glioblastoma | 3/182 | 1.65 | 0.53-4.98 | 2/182 | 1.10 | 0.28-4.29 |
| Mesothelioma | 17/300 | 5.67 | 3.55-8.93 | 7/300 | 2.33 | 1.12-4.81 |

ICI, immune checkpoint inhibitor; n/N refers to the number of events (n) observed for the outcome regarding the overall number of patients (N) in patients treated with immune checkpoint inhibitor therapy; CI, confidence interval. Chem, chemotherapy; Targeted, targeted therapy;NSCLC, non-small cell lung cancer; SCLC, small cell lung cancer; GEJC, gastroesophageal junction cancer; UC, urothelial carcinoma; RCC, renal cell carcinoma; BC, breast cancer; HNSCC, head and neck squamous cell carcinoma; PC, prostate cancer; HCC, hepatocellular carcinoma; ESO, esophageal carcinoma; OC, ovarian cancer; CRC, colorectal cancer.
