# Supplementary material for: Pancreatic adverse events of immune checkpoint inhibitors therapy for solid cancer patients: a systematic review and meta-analysis
Source: Front Immunol. 2023 Jun 9;14:1166299. doi: 10.3389/fimmu.2023.1166299 (PMC10289552; doi:10.3389/fimmu.2023.1166299)
Supplement: Supplementary file 5 [file Table_5.docx]

| Supplementary Table 5. Incidences of ICI therapy-associated lipase elevation in randomized controlled trials. | | | | | | |
| --- | --- | --- | --- | --- | --- | --- |
| Variables | **Lipase Elevation** | | | | | |
|  | **Grade 1-5** | | | **Grade 3-5** | | |
|  | **n/N** | **Incidence (%)** | **95%CI** | **n/N** | **Incidence**  **(%)** | **95%CI** |
| Combination type |  | | | | | |
| Single ICI therapy | 88/5281 | 1.45 | 0.88-2.38 | 50/5851 | 1.01 | 0.64-1.57 |
| ICI+ Chem/Targeted | 312/4168 | 5.34 | 2.70-10.29 | 156/4517 | 2.23 | 1.10-4.49 |
| Dual ICI therapy | 188/2959 | 4.08 | 1.70-9.49 | 130/2959 | 3.28 | 1.53-6.87 |
| Cancer type |  | | | | | |
| NSCLC | 54/3505 | 1.15 | 0.52-2.50 | 30/3503 | 0.69 | 0.30-1.57 |
| SCLC | 23/1009 | 1.94 | 0.35-9.90 | 16/1009 | 1.52 | 0.37-6.06 |
| Melanoma | 98/843 | 9.28 | 1.23-45.57 | 64/843 | 6.14 | 0.88-32.55 |
| GEJC | 99/1209 | 3.63 | 0.73-16.17 | 48/1209 | 1.70 | 0.35-7.75 |
| UC | 70/1426 | 4.20 | 1.83-9.34 | 46/2077 | 1.91 | 0.82-4.38 |
| RCC | 144/1257 | 5.18 | 0.38-43.99 | 77/1257 | 3.18 | 0.39-21.35 |
| BC | - | - | - | - | - | - |
| HNSCC | 10/348 | 2.87 | 1.55-5.26 | 5/348 | 1.44 | 0.60-3.40 |
| PC | 2/393 | 0.51 | 0.13-2.01 | 1/393 | 0.25 | 0.04-1.78 |
| HCC | 28/429 | 6.53 | 4.38-9.30 | 7/429 | 1.63 | 0.66-3.33 |
| ESO | - | - | - | - | - | - |
| OC | 22/1026 | 2.03 | 1.04-3.92 | 16/1026 | 1.58 | 0.85-2.89 |
| CRC | 10/269 | 3.15 | 0.79-11.76 | 5/269 | 1.94 | 0.81-4.58 |
| Glioblastoma | 7/182 | 3.85 | 1.84-7.85 | 4/182 | 2.20 | 0.83-5.71 |
| Mesothelioma | 21/521 | 2.10 | 0.15-23.77 | 14/521 | 1.72 | 0.19-13.89 |

ICI, immune checkpoint inhibitor; n/N refers to the number of events (n) observed for the outcome regarding the overall number of patients (N) in patients treated with immune checkpoint inhibitor therapy; CI, confidence interval. Chem, chemotherapy; Targeted, targeted therapy; NSCLC, non-small cell lung cancer; SCLC, small cell lung cancer; GEJC, gastroesophageal junction cancer; UC, urothelial carcinoma; RCC, renal cell carcinoma; BC, breast cancer; HNSCC, head and neck squamous cell carcinoma; PC, prostate cancer; HCC, hepatocellular carcinoma; ESO, esophageal carcinoma; OC, ovarian cancer; CRC, colorectal cancer.
