# Supplementary material for: Pancreatic adverse events of immune checkpoint inhibitors therapy for solid cancer patients: a systematic review and meta-analysis
Source: Front Immunol. 2023 Jun 9;14:1166299. doi: 10.3389/fimmu.2023.1166299 (PMC10289552; doi:10.3389/fimmu.2023.1166299)
Supplement: Supplementary file 6 [file Table_6.docx]

| Supplementary Table 6. Summary pooled analysis on the risk of ICI therapy-associated pancreatitis vs. controls in randomized controlled trials. | | | | | | | |
| --- | --- | --- | --- | --- | --- | --- | --- |
| Variables | **Pancreatitis** | | | | | | |
|  | **Grade 1-5** | | | | **Grade 3-5** | | |
|  | **OR** | | **95%CI** | **P** | **OR** | **95%CI** | **P** |
| Combination type |  | | | | | | |
| Single ICI therapy | 1.80 | 0.92-3.51 | | 0.09 | 1.57 | 0.63-3.94 | 0.34 |
| ICI+ Chem/Targeted | 2.00 | 1.25-3.22 | | 0.004 | 1.30 | 0.69-2.42 | 0.42 |
| Dual ICI therapy | 3.47 | 1.22-9.91 | | 0.02 | 3.56 | 1.09-11.65 | 0.04 |
| Cancer type |  | | | | | | |
| NSCLC | 2.55 | | 1.32-4.92 | 0.005 | 1.76 | 0.71-4.33 | 0.22 |
| SCLC | 3.62 | | 0.59-22.31 | 0.16 | 3.02 | 0.47-19.19 | 0.24 |
| Melanoma | 4.92 | | 1.43-16.95 | 0.01 | 4.91 | 0.84-28.83 | 0.08 |
| GEJC | 0.74 | | 0.14-3.86 | 0.72 | 0.31 | 0.01-7.69 | 0.48 |
| UC | 1.70 | | 0.63-4.58 | 0.30 | 1.61 | 0.48-5.42 | 0.44 |
| RCC | 1.48 | | 0.41-5.32 | 0.54 | 1.03 | 0.26-4.01 | 0.97 |
| BC | - | | - | - | - | - | - |
| HNSCC | 3.93 | | 0.43-35.65 | 0.22 | 3.13 | 0.13-77.18 | 0.49 |
| PC | - | | - | - | - | - | - |
| HCC | 0.99 | | 0.41-2.38 | 0.98 | 1.15 | 0.07-19.93 | 0.92 |
| ESO | 2.29 | | 0.33-15.70 | 0.40 | 0.98 | 0.10-9.47 | 0.99 |
| OC | 4.55 | | 0.53-39.04 | 0.17 | 4.04 | 0.47-35.03 | 0.20 |
| CRC | 2.46 | | 0.27-22.48 | 0.42 | 2.46 | 0.27-22.48 | 0.42 |
| Glioblastoma | - | | - | - | - | - | - |
| Mesothelioma | 2.77 | | 0.31-25.20 | 0.37 | - | - | - |

ICI, immune checkpoint inhibitor; CI, confidence interval; OR, odds ratio. Chem, chemotherapy; Targeted, targeted therapy. NSCLC, non-small cell lung cancer; SCLC, small cell lung cancer; GEJC, gastroesophageal junction cancer; UC, urothelial carcinoma; RCC, renal cell carcinoma; BC, breast cancer; HNSCC, head and neck squamous cell carcinoma; PC, prostate cancer; HCC, hepatocellular carcinoma; ESO, esophageal carcinoma; OC, ovarian cancer; CRC, colorectal cancer.
