# Supplementary material for: Pancreatic adverse events of immune checkpoint inhibitors therapy for solid cancer patients: a systematic review and meta-analysis
Source: Front Immunol. 2023 Jun 9;14:1166299. doi: 10.3389/fimmu.2023.1166299 (PMC10289552; doi:10.3389/fimmu.2023.1166299)
Supplement: Supplementary file 7 [file Table_7.docx]

| Supplementary Table 7. Summary pooled analysis on the risk of ICI therapy-associated amylase elevation vs. controls in randomized controlled trials. | | | | | | |
| --- | --- | --- | --- | --- | --- | --- |
| Variables | **Amylase Elevation** | | | | | |
|  | **Grade 1-5** | | | **Grade 3-5** | | |
|  | **OR** | **95%CI** | **P** | **OR** | **95%CI** | **P** |
| Combination type |  | | | | | |
| Single ICI agents | 1.86 | 1.28-2.69 | 0.001 | 1.83 | 0.99-3.38 | 0.05 |
| ICI+ Chem/Targeted | 1.60 | 1.09-2.35 | 0.02 | 1.75 | 1.14-2.69 | 0.01 |
| Dual ICI agents | 3.79 | 1.68-8.57 | 0.001 | 7.89 | 2.64-23.54 | 0.0002 |
| Cancer type |  | | | | | |
| NSCLC | 1.98 | 0.94-4.19 | 0.07 | 2.17 | 0.83-5.68 | 0.11 |
| SCLC | 4.10 | 1.44-11.63 | 0.008 | 3.10 | 0.55-17.66 | 0.20 |
| Melanoma | 1.30 | 0.88-1.92 | 0.19 | 1.81 | 0.84-3.94 | 0.13 |
| GEJC | 0.76 | 0.18-3.14 | 0.70 | 0.45 | 0.10-2.01 | 0.29 |
| UC | 4.64 | 1.30-16.49 | 0.02 | 4.29 | 1.84-10.02 | 0.0008 |
| RCC | 1.71 | 1.06-2.74 | 0.03 | 1.35 | 0.55-3.33 | 0.52 |
| BC | - | - | - | - | - | - |
| HNSCC | 4.00 | 1.55-10.33 | 0.004 | 2.97 | 0.71-12.51 | 0.14 |
| PC | 2.02 | 0.18-22.37 | 0.57 | 5.06 | 0.24-105.82 | 0.30 |
| HCC | 1.64 | 0.85-3.18 | 0.14 | 2.77 | 0.29-26.78 | 0.38 |
| ESO | - | - | - | - | - | - |
| OC | 2.21 | 0.89-5.53 | 0.09 | 1.08 | 0.24-4.91 | 0.93 |
| CRC | 0.76 | 0.25-2.31 | 0.63 | 3.19 | 0.16-62.54 | 0.44 |
| Glioblastoma | 2.75 | 0.28-26.69 | 0.38 | 4.58 | 0.22-96.19 | 0.33 |
| Mesothelioma | 17.00 | 2.25-128.60 | 0.006 | 14.54 | 0.83-255.76 | 0.07 |

ICI, immune checkpoint inhibitor; CI, confidence interval; OR, odds ratio. Chem, chemotherapy; Targeted, targeted therapy. NSCLC, non-small cell lung cancer; SCLC, small cell lung cancer; GEJC, gastroesophageal junction cancer; UC, urothelial carcinoma; RCC, renal cell carcinoma; BC, breast cancer; HNSCC, head and neck squamous cell carcinoma; PC, prostate cancer; HCC, hepatocellular carcinoma; ESO, esophageal carcinoma; OC, ovarian cancer; CRC, colorectal cancer.
