# Supplementary material for: Pancreatic adverse events of immune checkpoint inhibitors therapy for solid cancer patients: a systematic review and meta-analysis
Source: Front Immunol. 2023 Jun 9;14:1166299. doi: 10.3389/fimmu.2023.1166299 (PMC10289552; doi:10.3389/fimmu.2023.1166299)
Supplement: Supplementary file 8 [file Table_8.docx]

| Supplementary Table 8. Summary pooled analysis on the risk of ICI therapy-associated lipase elevation vs. controls in randomized controlled trials. | | | | | | |
| --- | --- | --- | --- | --- | --- | --- |
| Variables | **Lipase Elevation** | | | | | |
|  | **Grade 1-5** | | | **Grade 3-5** | | |
|  | **OR** | **95%CI** | **P** | **OR** | **95%CI** | **P** |
| Combination type |  | | | | | |
| Single ICI agents | 1.41 | 0.81-2.47 | 0.22 | 1.55 | 0.94-2.55 | 0.08 |
| ICI+ Chem/Targeted | 1.72 | 1.34-2.20 | ＜0.0001 | 1.63 | 1.21-2.21 | 0.002 |
| Dual ICI agents | 2.92 | 1.37-6.20 | 0.005 | 3.36 | 1.64-6.88 | 0.0009 |
| Cancer type |  | | | | | |
| NSCLC | 4.23 | 2.14-8.34 | ＜0.0001 | 3.64 | 1.55-8.51 | 0.003 |
| SCLC | 1.64 | 0.84-3.20 | 0.14 | 1.95 | 0.84-4.53 | 0.12 |
| Melanoma | 1.09 | 0.80-1.50 | 0.58 | 1.15 | 0.79-1.68 | 0.47 |
| GEJC | 1.14 | 0.31-4.15 | 0.84 | 0.85 | 0.15-4.99 | 0.86 |
| UC | 4.20 | 1.46-12.09 | 0.008 | 3.79 | 1.64-8.74 | 0.002 |
| RCC | 1.53 | 1.16-2.01 | 0.003 | 1.50 | 1.04-2.17 | 0.03 |
| BC | - | - | - | - | - | - |
| HNSCC | 2.01 | 0.68-5.93 | 0.21 | 5.00 | 0.58-43.02 | 0.14 |
| PC | 1.01 | 0.14-7.19 | 0.99 | 0.50 | 0.05-5.56 | 0.57 |
| HCC | 1.90 | 0.99-3.66 | 0.06 | 1.29 | 0.41-4.11 | 0.66 |
| ESO | - | - | - | - | - | - |
| OC | 3.42 | 1.17-9.97 | 0.02 | 3.49 | 1.01-12.02 | 0.05 |
| CRC | 0.40 | 0.09-1.68 | 0.21 | 1.37 | 0.24-7.75 | 0.72 |
| Glioblastoma | 6.56 | 0.80-53.90 | 0.08 | 8.34 | 0.45-156.18 | 0.16 |
| Mesothelioma | 7.46 | 0.59-93.67 | 0.12 | 6.33 | 0.86-46.81 | 0.07 |

ICI, immune checkpoint inhibitor; CI, confidence interval; OR, odds ratio. Chem, chemotherapy; Targeted, targeted therapy. NSCLC, non-small cell lung cancer; SCLC, small cell lung cancer; GEJC, gastroesophageal junction cancer; UC, urothelial carcinoma; RCC, renal cell carcinoma; BC, breast cancer; HNSCC, head and neck squamous cell carcinoma; PC, prostate cancer; HCC, hepatocellular carcinoma; ESO, esophageal carcinoma; OC, ovarian cancer; CRC, colorectal cancer.
