# Supplementary material for: Pancreatic adverse events of immune checkpoint inhibitors therapy for solid cancer patients: a systematic review and meta-analysis
Source: Front Immunol. 2023 Jun 9;14:1166299. doi: 10.3389/fimmu.2023.1166299 (PMC10289552; doi:10.3389/fimmu.2023.1166299)
Supplement: Supplementary file 9 [file Table_9.docx]

| Supplementary Table 9. Heterogeneity in this meta-analysis | | | | |
| --- | --- | --- | --- | --- |
|  | **Subgroups** | **N．arms** | **I²** | **P** |
| Pancreatitis（G1-5） | | | | |
| Caner type | | | | |
|  | NSCLC | 18 | 0 | 0.98 |
|  | SCLC | 3 | 0 | 0.97 |
|  | Melanoma | 5 | 0 | 0.79 |
|  | GEJC | 3 | 0 | 0.56 |
|  | UC | 5 | 0 | 0.62 |
|  | RCC | 2 | - | - |
|  | BC | 1 | - | - |
|  | HNSCC | 2 | 0 | 0.85 |
|  | PC | 0 | - | - |
|  | HCC | 2 | - | - |
|  | ESO | 2 | 0 | 0.86 |
|  | OC | 2 | 0 | 0.36 |
|  | CRC | 2 | 0 | 0.94 |
|  | Mesothelioma | 2 | 0 | 0.61 |
|  | Glioblastoma | 0 | - | - |
|  | Total | 49 | 0 | 1.00 |
| Combination type | | | | |
|  | Single ICI agents | 19 | 0 | 0.96 |
|  | ICI+ Chem/Targeted | 24 | 0 | 0.99 |
|  | Dual ICI agents | 6 | 0 | 0.96 |
|  | Total | 49 | 0 | 1.00 |
| Pancreatitis（G3-5） | | | | |
| Caner type | | | | |
|  | NSCLC | 16 | 0 | 0.82 |
|  | SCLC | 3 | 0 | 1.00 |
|  | Melanoma | 4 | 0 | 0.61 |
|  | GEJC | 1 | - | - |
|  | UC | 5 | 0 | 0.50 |
|  | RCC | 2 | - | - |
|  | BC | 1 | - | - |
|  | HNSCC | 2 | - | - |
|  | PC | 0 | - | - |
|  | HCC | 2 | - | - |
|  | ESO | 2 | 0 | 0.35 |
|  | OC | 2 | 0 | 0.41 |
|  | CRC | 2 | 0 | 0.94 |
|  | Mesothelioma | 1 | - | - |
|  | Glioblastoma | 0 | - | - |
|  | Total | 43 | 0 | 0.99 |
| Combination type | | | | |
|  | Single ICI agents | 16 | 0 | 0.67 |
|  | ICI+ Chem/Targeted | 20 | 0 | 0.97 |
|  | Dual ICI agents | 7 | 0 | 0.93 |
|  | Total | 43 | 0 | 0.99 |
| Amylase Elevation（G1-5） | | | | |
| Caner type | | | | |
|  | NSCLC | 13 | 59% | 0.01 |
|  | SCLC | 3 | 0 | 0.84 |
|  | Melanoma | 3 | - | - |
|  | GEJC | 2 | 42% | 0.19 |
|  | UC | 4 | 54% | 0.02 |
|  | RCC | 2 | - | - |
|  | BC | 0 | - | - |
|  | HNSCC | 2 | - | - |
|  | PC | 1 | - | - |
|  | HCC | 2 | - | - |
|  | ESO | 0 | - | - |
|  | OC | 3 | 0 | 0.66 |
|  | CRC | 2 | 0 | 0.72 |
|  | Mesothelioma | 1 | - | - |
|  | Glioblastoma | 1 | - | - |
|  | Total | 39 | 36% | 0.03 |
| Combination type | | | | |
|  | Single ICI agents | 17 | 0 | 0.68 |
|  | ICI+ Chem/Targeted | 15 | 51% | 0.02 |
|  | Dual ICI agents | 7 | 0 | 0.70 |
|  | Total | 39 | 36% | 0.03 |
| Amylase Elevation（G3-5） | | | | |
| Caner type | | | | |
|  | NSCLC | 13 | 0 | 0.47 |
|  | SCLC | 3 | 5% | 0.30 |
|  | Melanoma | 3 | - | - |
|  | GEJC | 2 | 0 | 0.84 |
|  | UC | 6 | 0 | 0.45 |
|  | RCC | 2 | - | - |
|  | BC | 0 | - | - |
|  | HNSCC | 2 | 0 | 0.98 |
|  | PC | 1 | - | - |
|  | HCC | 1 | - | - |
|  | ESO | 0 | - | - |
|  | OC | 3 | 0 | 0.64 |
|  | CRC | 2 | - | - |
|  | Mesothelioma | 1 | - | - |
|  | Glioblastoma | 1 | - | - |
|  | Total | 40 | 0 | 0.55 |
| Combination type | | | | |
|  | Single ICI agents | 18 | 0 | 0.85 |
|  | ICI+ Chem/Targeted | 15 | 0 | 0.44 |
|  | Dual ICI agents | 7 | 0 | 0.52 |
|  | Total | 40 | 0 | 0.55 |
| Lipase Elevation(G1-5) | | | | |
| Caner type | | | | |
|  | NSCLC | 11 | 0 | 0.90 |
|  | SCLC | 3 | 0 | 0.90 |
|  | Melanoma | 3 | - | - |
|  | GEJC | 3 | 82% | 0.004 |
|  | UC | 4 | 57% | 0.07 |
|  | RCC | 3 | 0 | 0.75 |
|  | BC | 0 | - | - |
|  | HNSCC | 1 | - | - |
|  | PC | 1 | - | - |
|  | HCC | 1 | - | - |
|  | ESO | 0 | - | - |
|  | OC | 3 | 0 | 0.88 |
|  | CRC | 2 | 40% | 0.20 |
|  | Mesothelioma | 2 | 47% | 0.17 |
|  | Glioblastoma | 1 | - | - |
|  | Total | 38 | 46% | 0.004 |
| Combination type | | | | |
|  | Single ICI agents | 18 | 36% | 0.09 |
|  | ICI+ Chem/Targeted | 12 | 33% | 0.14 |
|  | Dual ICI agents | 8 | 72% | 0.003 |
|  | Total | 38 | 46% | 0.004 |
| Lipase Elevation(G3-5) | | | | |
| Caner type | | | | |
|  | NSCLC | 11 | 0 | 0.88 |
|  | SCLC | 3 | 0 | 0.86 |
|  | Melanoma | 4 | 0 | 0.90 |
|  | GEJC | 3 | 78% | 0.01 |
|  | UC | 6 | 14% | 0.33 |
|  | RCC | 3 | 0 | 0.66 |
|  | BC | 0 | - | - |
|  | HNSCC | 1 | - | - |
|  | PC | 1 | - | - |
|  | HCC | 1 | - | - |
|  | ESO | 0 | - | - |
|  | OC | 3 | 0 | 0.97 |
|  | CRC | 2 | 0 | 0.69 |
|  | Mesothelioma | 2 | 20% | 0.26 |
|  | Glioblastoma | 1 | - | - |
|  | Total | 41 | 26% | 0.10 |
| Combination type | | | | |
|  | Single ICI agents | 20 | 0 | 0.53 |
|  | ICI+ Chem/Targeted | 13 | 21% | 0.25 |
|  | Dual ICI agents | 8 | 64% | 0.02 |
|  | Total | 41 | 26% | 0.10 |

Subgroup analyses on the summary risk of pancreatic adverse events based on the underlying cancer type and the combination type. NSCLC, non-small cell lung cancer; SCLC, small cell lung cancer; GEJC, gastroesophageal junction cancer; UC, urothelial carcinoma; RCC, renal cell carcinoma; BC, breast cancer; HNSCC, head and neck squamous cell carcinoma; PC, prostate cancer; HCC, hepatocellular carcinoma; ESO, esophageal carcinoma; OC, ovarian cancer; CRC, colorectal cancer; Chem, chemotherapy; Targeted, targeted therapy.
