# Supplementary material for: Pancreatic adverse events of immune checkpoint inhibitors therapy for solid cancer patients: a systematic review and meta-analysis
Source: Front Immunol. 2023 Jun 9;14:1166299. doi: 10.3389/fimmu.2023.1166299 (PMC10289552; doi:10.3389/fimmu.2023.1166299)
Supplement: Supplementary file 10 [file Table_10.doc]

**Supplementary Table 10. Funnel plot and Peter’s test**

**
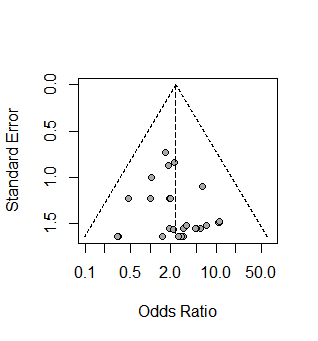

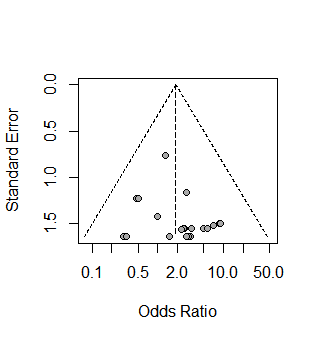
**

**Pancreatitis G1-5 Pancreatitis G3-5**

**P= 0.49 P= 0.96**

**
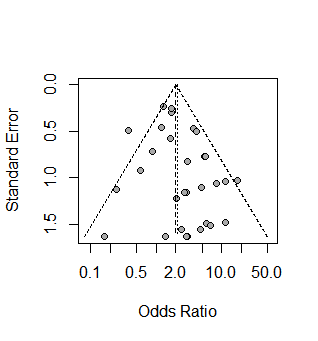

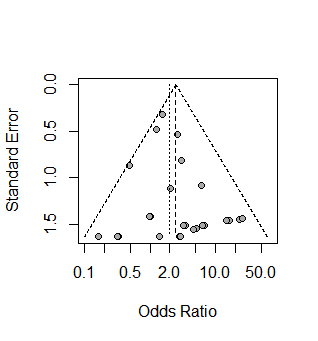
**

**Amylase elevation G1-5 Amylase elevation G3-5**

**P= 0.13 P= 0.18**

**
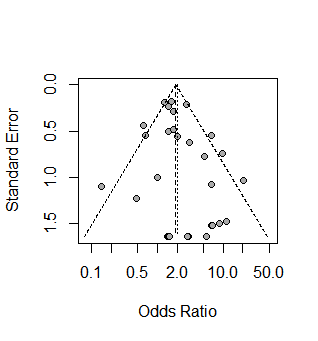

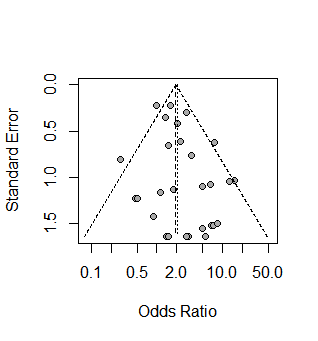
**

**Lipase elevation G1-5 Lipase elevation G3-5**

**P= 0.05 P= 0.30**
